# Laser-emission vibrational microscopy of microdroplet arrays for high-throughput screening of hyperlipidemia

**DOI:** 10.1038/s41377-025-02015-5

**Published:** 2025-09-17

**Authors:** Zhonghao Li, Zhihan Cai, Yuhan Wang, Yuliang Liu, Guifeng Li, Xi Yang, Ming Deng, Yu-Cheng Chen, Jichun Yang, Yang Luo, Chaoyang Gong, Tao Zhu

**Affiliations:** 1 Key Laboratory of Optoelectronic Technology and Systems (Ministry of Education of China), School of Optoelectronic Engineering, Chongqing University, Chongqing, 400044 China; 2 Department of Laboratory Medicine, Chongqing Center for Clinical Laboratory, Chongqing Academy of Medical Sciences, Chongqing General Hospital, School of Medicine, Chongqing University, Chongqing, 401147 China; 3 Key Laboratory of Optical Fiber Sensing and Communications (Ministry of Education of China), School of Information and Communication Engineering, University of Electronic Science and Technology of China, Chengdu, Sichuan 611731 China; 4 School of Electrical and Electronic Engineering, Nanyang Technological University, Singapore, 639798 Singapore; 5 Lee Kong Chian School of Medicine, Singapore, 636921, Singapore

**Keywords:** Imaging and sensing, Microresonators

## Abstract

The mechanical properties of biological fluids serve as early indicators of disease, offering valuable insights into complex physiological and pathological processes. However, the existing technologies struggle to achieve high-throughput measurement, limiting their widespread applications in disease diagnosis. Here, we propose laser-emission vibrational microscopy of microdroplets for high-throughput measurement of the intrinsic mechanical properties of fluids. The microdroplet array supporting high Q-factor (10^4^) whispering gallery modes (WGM) lasing was massively fabricated on a superhydrophobic surface with inkjet printing. Ultrasound was employed to actuate the mechanical vibrations of the microdroplets, and the vibration amplitude was quantified using time-resolved laser spectra. We found that the stimulus-response of the laser emission is strongly dependent on the liquid viscosity. Fast mapping of the microdroplets’ viscosities was achieved by stage scanning. High-throughput screening of hyperlipidemia disease was also demonstrated by performing over 2000 measurements within 25 min. Thanks to the small volume of the microdroplets, a single drop of blood can support over seven million measurements. The high-throughput ability and small sample consumption make it a promising tool for clinical diagnoses based on mechanical properties.

## Abstract (web summary)

Laser-emission vibrational microscopy quantifies liquid viscosity with whispering gallery mode lasers, enabling high-throughput screening of hyperlipidemia disease.

## Graphical abstract

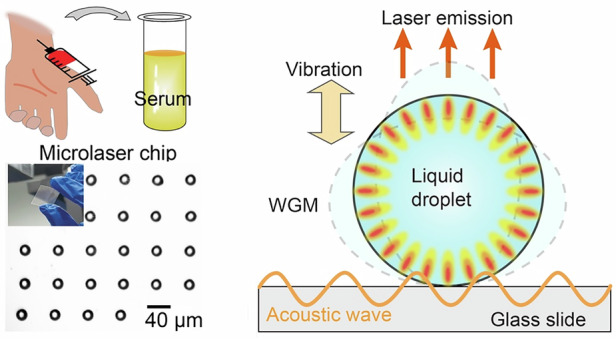

## Introduction

Biological fluid analysis serves as the foundation for diagnostics and clinical decision-making across a broad range of pathologies^1–3^. The mechanical properties of biological fluids, such as viscosity and surface tension, can serve as early indicators of disease, providing rich information in understanding the complex physiological and pathological processes^4,5^. For example, alterations in blood viscosity are associated with cardiovascular diseases and can precede detectable biochemical changes^6^. Similarly, the surface tension of fluids such as serum and saliva has been linked to conditions like cancer, where variations may signal changes in tumor microenvironments^7^. Techniques based on rheological measurements^8^, atomic force microscopy (AFM)^9^, Brillouin spectroscopy^10,11^, optical tweezers^12^, and microfluidics^13^ have been developed to assess the mechanical properties of biological fluid, driving the rapid expansion of mechanobiology. However, the low throughput of these existing technologies limits their potential for diagnostic applications. Therefore, developing high-throughput methodologies for extracting mechanical information from biological fluids is highly desirable to enhance diagnostic efficiency and expand biomedical applications.

Optical microcavities are recently emerging as a powerful tool to detect mechanical properties in biological systems^14–18^. Among them, the whispering gallery mode (WGM) microcavities have drawn much attention because their ultrahigh Q-factors provide significantly enhanced intracavity light-matter interaction^19–23^. The interaction between optical modes and mechanical stimulus enables precisely quantifying essential mechanical parameters such as force^24^, pressure^25^, viscosity^26^, and vibration frequency^27,28^. In these configurations, near-field excitation of high Q-factor WGMs is required to achieve high sensitivity, which compromises system stability and restricts high-throughput measurements. In contrast, the WGM microlasers, which integrate gain medium within WGM microcavities, enable free-space excitation and signal collection^29–34^. By leveraging their far-field coupling capabilities, WGM microlasers can function as freely dispersible probes in diverse biological environments, enabling intracellular^35^ and deep-tissue^36^ measurements. However, due to their inability to apply stimulus to the external environment, current WGM microlasers fall short in revealing the intrinsic mechanical properties of biological samples.

**Fig. 1:**
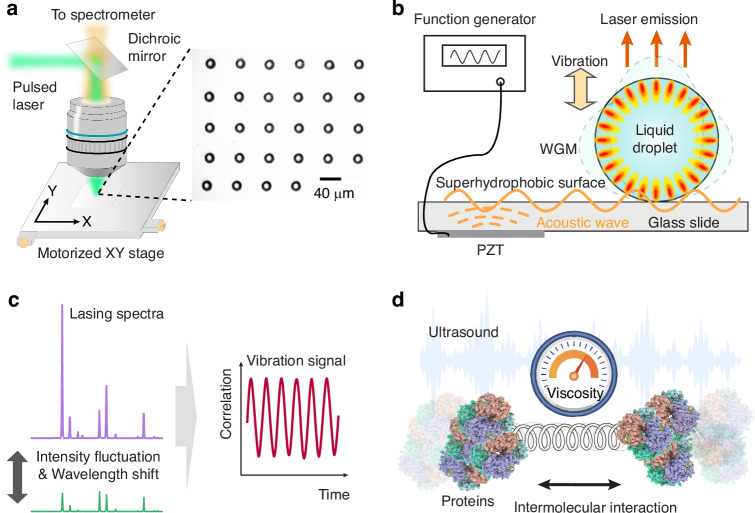
**The principle of laser-emission vibrational microscopy**. **a** Illustration of the high-throughput scanning of the microdroplet laser array. **b** Schematic illustration of the coupling between ultrasound and WGMs. **c** Extraction of vibration signal from laser spectra. **d** Illustration of revealing molecular interactions with ultrasound

Here, we propose the concept of laser-emission vibrational microscopy (LEVM) of microdroplets, which utilizes ultrasound to stimulate microdroplet lasers, and the mechanical vibrational response was detected with WGM laser emission. As illustrated in Fig. 1a, the dye-filled microdroplet array was fabricated on a superhydrophobic surface with a commercial inkjet printer and was excited in free space with a pulsed laser. The spherical microdroplet supports high-Q factor WGM, providing strong optical feedback for lasing. The ultrasound applied to the liquid droplet induces vibrations, resulting in periodic intensity fluctuations and wavelength shifts of laser emission (Fig. 1b, c). Our findings indicate that the vibrational behavior obtained from laser emission strongly depends on the liquid’s intrinsic viscosity, offering mechanical insights into molecular interactions (Fig. 1d). Thanks to the efficient far-field excitation and signal collection of the WGM laser, high-throughput mechanical mapping of the microdroplets made from hyperlipidemia patients’ serum was demonstrated, highlighting its significant potential in clinical applications. Our work offers a high-throughput solution for quantifying molecular interactions in liquids, paving the way for clinical diagnosis based on mechanical properties.

## Results

### Coupling mechanism of ultrasound and microdroplet laser

**Fig. 2:**
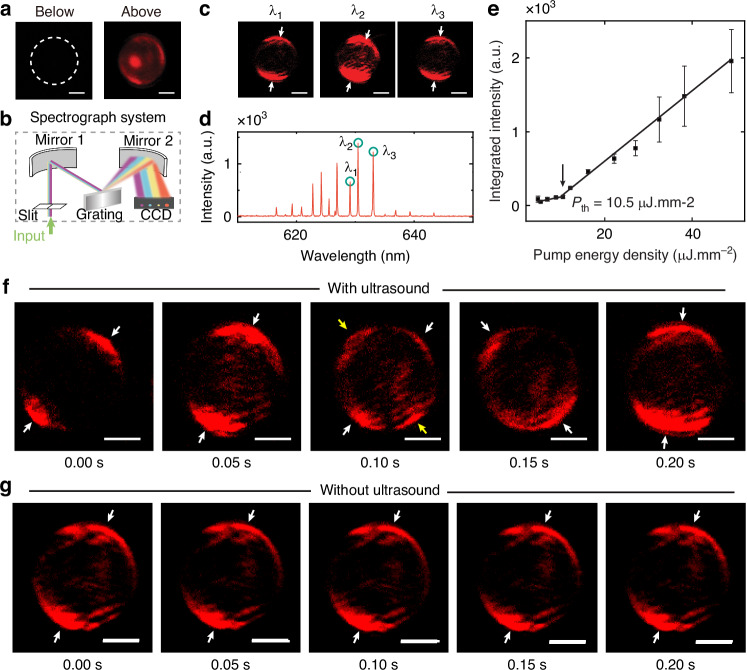
**Coupling mechanism between ultrasound and microdroplet laser**. **a** Image of the microdroplet with the pump energy density below (**left**) and above (**right**) the lasing threshold. The dashed circle indicates the boundary of the microdroplet. Scale bar: 5 µm. **b** Illustration of the spectrograph system. The laser pattern was dispersed by a diffractive grating according to its spectral components. **c** Spectral components of the laser pattern in (**a**). Scale bar: 5 µm. **d** Laser emission spectrum of the microdroplet in (**a**). **e** The spectrally integrated intensity as a function of various pump energy densities. Error bars are obtained based on triplicate measurements. Time-resolved laser spectral images with (**f**) and without (**g**) ultrasound excitation. The arrows indicate the orientations of the WGMs. Images are recorded at 629.1 nm. Scale bar: 5 µm

An upright microscope system was used for laser excitation and signal collection (Supplementary Fig. S1). The microdroplets with a diameter of 20 μm were massively fabricated on a superhydrophobic surface by a commercial inkjet printer, forming a contact angle larger than 130° (Supplementary Fig. S2). The microdroplets with nearly spherical morphology support WGMs with high Q-factors of up to 6$\times$10^4^ (See Supplementary Materials for more details), providing strong optical feedback for lasing. As illustrated in Fig. 2a, due to the tangential radiation of the WGM, bright laser emission started appearing at the edge of the microdroplet after the pump energy density was above the lasing threshold. The observed laser pattern of the liquid droplet can be regarded as the superposition of spatial modes with different lasing wavelengths. To visualize the components of the laser modes, we imaged the laser pattern with a spectrograph system (Fig. 2b). Due to the dispersive nature of the spectrograph system, the laser mode pattern is resolved based on its wavelength (Fig. 2c and Supplementary Fig. S3), allowing for the identification of mode components with slight phase differences. Unlike the Laguerre-Gaussian modes observed in Fabry-Perot (FP) cavities^37,38^, the laser modes of a WGM cavity consist of two symmetrically crescent-shaped components, the orientation of which is determined by the optical oscillation within the droplet (Fig. 2c). The laser spectrum is shown in Fig. 2d, with individual strong peaks corresponding to the longitudinal laser modes. The laser spectrum of the microdroplets remained stable over time, while relatively stronger mode competition was observed in larger droplets (Fig. S4). To achieve higher laser stability, the droplet size was kept at 20 µm throughout the experiment. Because of the high Q-factor of the liquid droplet, a low lasing threshold of about 10.5 μJ.mm-2 was obtained (Fig. 2e), which is comparable to the other microdroplet lasers^39,40^.

Then, we investigated the coupling mechanism of ultrasound and microdroplet lasers by monitoring the temporal evolution of spectral images. As shown in Fig. 2f, the spectral images at different observation times exhibited significantly different orientations, which can be explained by the periodic mechanical vibration actuated by ultrasound. In contrast, the spectral images remain stable in the absence of ultrasound (Fig. 2g). As illustrated in Fig. 1b, the ultrasound exerted a time-varying force on the microdroplet, inducing a periodic deformation in morphology. When the microdroplet changes from spherical to elliptical, the Q-factor of the WGM drops due to chaotic resonance (Supplementary Fig. S5). As a result, only WGMs with higher Q-factors support lasing in mode competition. The divergence of the oscillation direction reveals the asymmetrical geometry of the microcavity, which offers a potential approach for measuring the anisotropy of mechanical forces in the surrounding environment^41^.

### Quantification of mechanical vibration via laser spectrum

**Fig. 3:**
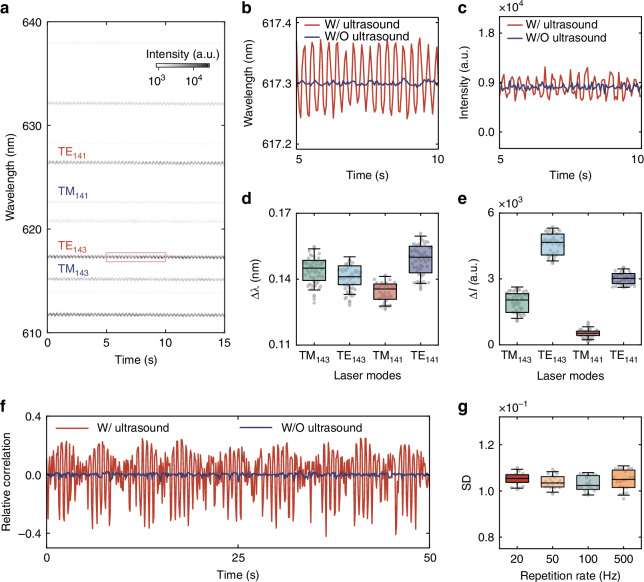
**Quantification of mechanical vibration with laser emission**. **a** Temporal evolution of lasing spectra induced by ultrasound. **b**, **c** Temporal evolution of wavelength and intensity of the lasing peak. Data are extracted from the boxed region in (**a**). Wavelength shifts (**d**) and intensity fluctuations (**e**) obtained from different orders of lasing modes. Data were extracted from the peak-to-peak values in (**b**, **c**), respectively. Error bars represent standard deviations of ~60 measurements. **f** Comparison of time-varying relative correlation curves with (**red**) and without (**blue**) ultrasound. **g** Standard division of the relative correlation curves with different pump repetition rates. Error bars represent standard deviations of 20 measurements

The morphological changes in microdroplet alter the periodic switching of WGMs oscillation direction, resulting in a significant wavelength shift of laser peaks (Fig. 3a). Figure 3b shows the enlargement of the periodic wavelength shift. A maximum shift of approximately 0.08 nm was observed, corresponding to a small geometric deformation of 6.5 nm^42^ (Supplementary Fig. S6). This value is significantly below the diffraction limit of the microscope (~573 nm), which confirms the extraordinary ability of the WGM laser in measuring subtle mechanical vibrations. Furthermore, due to the varying intracavity losses encountered by the WGMs in different orientations^43,44^, intensity fluctuation in the lasing peak was also observed (Fig. 3c)^45,46^. Interestingly, distinct wavelength shifts and intensity fluctuations are observed in WGMs with different orders (Fig. 3d, e), which is due to the spatial heterogeneity of WGMs on the microdroplet. This relatively large divergence hinders the accurate quantification of mechanical vibrations by relying on the conventional methods that track the wavelength shift or intensity fluctuation of a single peak^47^.

To overcome this issue, we calculated the temporal correlation of laser spectra, incorporating the lasing wavelength shifts and intensity variations in the analysis (see Methods for details). As illustrated in Fig. 3f, the temporal relative correlation curve is obtained by subtracting the smooth curve from the temporal correlation curve (Supplementary Fig. S7), which exhibits a fast fluctuation superimposed on a slow envelope. The fast Fourier transform (FFT) spectrum has a sharp peak at *f*_1_ = 0.3 Hz, which shows an identical peak location and a higher signal-to-noise ratio compared to the FFT spectra of wavelength shifts and intensity variations (Supplementary Fig. S8). This result indicates that the temporal relative correlation accurately captures the features of the laser spectra evolution. We have to note that the peak frequency observed in the FFT spectrum is six orders of magnitude lower than the ultrasound excitation (132.4003 kHz). This is owing to the under sampling effect caused by the low pump repetition rate (20 Hz) (Supplementary Figs. S9–S10)^48^. According to the simulation (Fig. S11) and experimental (Fig. 3g) results, the standard division (SD) of the under sampling signal remains consistent with the original signal, which was employed as the sensing output throughout the experiment.

### Measuring liquid viscosity with laser emission

**Fig. 4:**
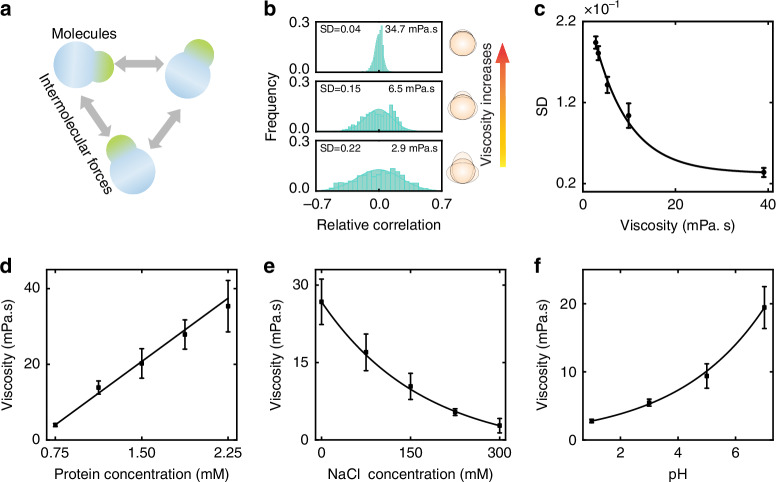
**Viscosity of BSA solution**. **a** Schematic illustration of molecular interaction. **b** Statistics distribution of the relative correlation with various glycerol concentrations. **c** Calibration curve. Error bars represent standard deviations of 10 measurements. Viscosity as a function of protein concentration (**d**), NaCl concentration (**e**) and pH (**f**). Error bars represent standard deviations of 10 measurements

The ultrasound-actuated vibration of microdroplets was employed to measure the mechanical properties of the liquid^49^. Viscosity was used as an example because of its significance in disease diagnosis. As shown in Fig. 4a, the viscosity of a liquid arises from molecular interactions and reflects the intermolecular forces that resist its flow^50–55^. Since the mechanical vibration of microdroplets strongly depends on the driving voltage and droplet size (Supplementary Figs. S12 and S13), these parameters were kept consistent throughout the experiment. We fabricated microdroplet lasers with various viscosities by varying the concentration of glycerol (Supplementary Fig. S14) and investigated their vibrational response to ultrasound stimulation. As shown in Fig. 4b, the statistical distribution of the relative correlation follows a normal distribution. The viscosity of the liquid droplet has a significant impact on their vibration behavior. Because the stronger intermolecular forces in high viscosity liquid inhibit the mechanical vibration, a narrower statistical distribution was observed. We calibrated the SD of the temporal relative correlation curve with various viscosities in Fig. 4c, showing a maximum sensitivity in the linear range (2.8 to 10 mPa.s). This result indicates that the microdroplet lasers are appropriate for monitoring subtle mechanical changes in low viscosity liquids. According to the results in Supplementary Fig. S15 and Table S1, this calibration curve can be applied to different types of liquids, demonstrating its robustness in viscosity measurements.

The BSA was chosen as the model protein to demonstrate feasibility of viscosity measurement. BSA has an isoelectric point of about pH = 5.1 and carries a negative net charge under neutral conditions. As a result, the BSA molecules experience electrostatic repulsion forces from the nearby molecules, which suppresses the mobility of BSA molecules. As the BSA concentration increases, the electrostatic repulsion forces increase and lead to a higher viscosity. As illustrated in Fig. 4d, the viscosity of BSA solution shows a linear relationship with concentration in the range from 0.75 to 2.25 mM^56^. When NaCl was inducted into the BSA solution, the strong charge shielding effect of Na^+^ and Cl^-^ weakened the electrostatic forces between BSA molecules. Therefore, increasing the NaCl concentration led to a decrease in the viscosity of the BSA solution (Fig. 4e). As the pH decreases from neutral to acidic conditions, the viscosity of the BSA solution decreases^57^, which can be attributed to protein denaturation under acidic conditions (Fig. 4f).

### High-speed mechanical mapping of microdroplet array

**Fig. 5:**
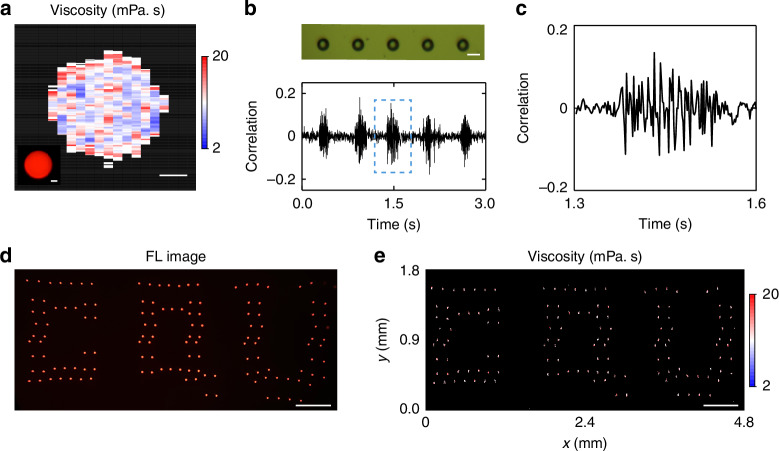
**Mapping viscosity of the microdroplet array**. **a** Viscosity map of a single microdroplet. Inset, the fluorescence image. Scale bar: 5 µm. **b** Bright-filed image of the microdroplet array (**top**) and the corresponding temporal relative correlation signal acquired through scanning (**bottom**). Scale bar: 20 µm. **c** Enlargement of the temporal relative correlation signal. Data are extracted from the boxed region in (**b**). Fluorescence image (**d**) and viscosity map (**e**) of the microdroplet array forming the character “CQU”. Scale bar: 500 µm

Stage scanning was employed to achieve high-throughput measurement of the mechanical vibrations of the microdroplets. In order to achieve a relatively fast scanning speed, a pulsed laser with a 500 Hz repetition rate was used for laser excitation. A viscosity map (Fig. 5a) of the microdroplet was generated by calculating the viscosity at each pixel by using the calibration curve in Fig. 4c (Supplementary Fig. S16). The variation is caused by a relatively small sample size in SD calculation, which was used as a trade-off between measurement accuracy and scanning speed. By increasing the sample size, the uniformity of viscosity map can be significantly improved (Supplementary Fig. S17). We also demonstrated that the diameter of pump spot and the scanning has negligible influence on the viscosity measurement (Supplementary Figs. S18–S20).

Massive production of microdroplet array was achieved by inkjet printing technology (See Methods for more details). The location and size of each droplet can be precisely controlled with the printer. As shown in Fig. 5b, c, the temporal relative correlation curve acquired with scanning contains the vibration signal of each droplet, enabling high-speed viscosity measurement of a droplet array. As illustrated in Fig. 5d, e, the viscosity map accurately reveals the mechanical information of each droplet. In this experiment, the scanning speed of the transition stage is set to 0.8 mm.s^−1^, allowing a 4.8 mm × 1.8 mm area to be scanned within 90 min. Assuming the area is printed with droplets at a separation of 40 μm, viscosities of up to 5400 droplets can be extracted. A higher scanning speed can be achieved by further increasing the pump repetition rate and the readout speed of the laser spectra. As illustrated Supplementary Fig. S21, the droplet size remains stable throughout the scanning process. These results indicate the great potential of LEVM in high-throughput measurement.

### High-throughput screening of hyperlipidemia

Hyperlipidemia is caused by high levels of fats in the blood and affects over 25 million people worldwide. It is diagnosed through elevated cholesterol levels, which increase blood viscosity and can lead to cardiovascular events such as strokes and heart disease^58,59^. The capability of LEVM in high-speed mechanical mapping of microdroplet array indicates its great potential for hyperlipidemia screening.

**Fig. 6:**
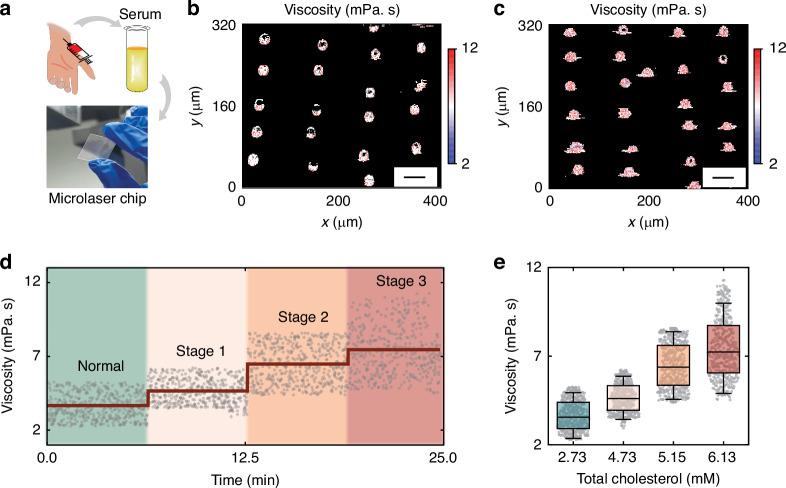
**High-throughput screening of hyperlipidemia**. **a** Illustration of fabricating serum microdroplet array. Viscosity maps of serum samples from a normal individual (**b**) and a hyperlipidemia patient (**c**). Scale bar: 40 µm. **d** High-throughput measurement of serum viscosities of four individuals. **e** Relationship between serum viscosities and total cholesterol levels in blood. Error bars represent standard deviations of ~500 measurements

The serum microdroplet array was used to demonstrate the potential of LEVM in clinical diagnosis (Fig. 6a). We compared the viscosity maps of serum samples from a normal individual and a hyperlipidemia patient in Fig. 6b, c, revealing significantly higher serum viscosity in the hyperlipidemia patient. We tested serum samples from four individuals, and the serum viscosities were clearly distinguished into four stages (normal stage, stage 1, stage 2, and stage 3) (Fig. 6d). As shown in Fig. 6e, the serum viscosities show a strong correlation with the total cholesterol levels in blood (Supplementary Table S2). The greater divergence of the results at higher cholesterol levels arises from the nonlinear nature of the calibration curve in Fig. 4c. The relatively low slope of the calibration curve at higher viscosities results in an increased error in viscosity measurements. In this experiment, high-throughput screening of over 2000 serum droplets were achieved within 25 min. Thanks to the small volume of the microdroplets (~4.2 pL), a single drop of blood (~30 μL) can support over seven million measurements. Further experiments in Supplementary Fig. S22 and Table S3 also confirm the strong potential of LEVM for clinical applications^54,60^.

## Discussion

We developed LEVM for rapid mechanical mapping of microdroplet arrays. The WGM laser emission from microdroplets was employed to detect the mechanical vibration induced by ultrasound. We explored the coupling mechanism between ultrasound and laser modes. The temporal behavior of laser emission induced by ultrasound was investigated and used to quantify the viscosity of the liquid. Rapid mechanical mapping of the microdroplets array was achieved by stage scanning, which was subsequently employed for high-throughput screening of hyperlipidemia.

Here, we would like to point out the significance of our work. First, we employed the optical microcavity as a new platform for high-throughput measuring of the intrinsic mechanical properties of biological fluids. Although current optical microcavities play an important role in assessing weak mechanical forces in biological fluids, most of them lack the ability to actively apply stimulus to the environment, making it difficult to detect the intrinsic mechanical properties of the biological system. The proposed stimulus-response measurement of microdroplet lasers offers a new approach to measure the intrinsic mechanical properties of biological fluids, which can unlock new possibilities in mechanical biomarkers discovery and low-cost clinical diagnosis. Second, this work provides a method for large-scale screening of cardiovascular risk. Serum viscosity plays a crucial role in the pathophysiology of various cardiovascular and metabolic disorders. For example, hyperlipidemia alters blood composition and rheological properties by increasing lipid levels, leading to elevated serum viscosity^61,62^. The proposed approach can facilitate the efficient identification of individuals with abnormal viscosity levels. When integrated with conventional lipid panel tests^63^, this method offers a more comprehensive evaluation of an individual’s cardiovascular health.

Finally, we discuss the advantage and disadvantage of the LEVM in hyperlipidemia diagnosis. Compared to conventional methods, the advantages of our approach are twofold. First, LEVM enables high-throughput detection of lipid-associated changes by probing the viscosity of serum samples, offering an indirect yet highly sensitive approach to detect lipid abnormalities. Second, the low cost and minimal sample requirement of make LEVM highly suitable for point-of-care applications and early-stage screening. However, unlike conventional enzymatic assays that provide direct quantification of lipid subtypes such as low-density and high-density lipoproteins^64^, LEVM relies on physical property measurements require additional models or calibration to establish accurate correlations with specific lipid concentrations^65^. Nonetheless, LEVM holds significant promise as a complementary diagnostic tool, particularly in situations demanding high sensitivity, high-throughput and low sample volume.

## Materials and methods

### Optical system setup

The experimental setup is illustrated in Supplementary Fig. S1. An upright microscope system equipped with a ×50 objective was used for microdroplet laser excitation and signal collection. An optical parametric oscillator (OPOTEK, MagicPRISM VIS), pumped by the third-harmonic wave of a Nd: YAG laser (Beamtech Optronics Co., Ltd., DAWA-200), served as the pump laser. The 532 nm pulsed pump laser (20 Hz repetition rate) was coupled into the upright microscope system and focused to a small spot with a diameter of 4 µm. The laser emission from the microdroplet was sent to a scientific complementary metal-oxide-semiconductor (sCMOS) camera (Tucsen, Dhyana 400BSI V3) and a spectrometer (Princeton Instruments, SpectrPro-500i) for image and spectral acquisition, respectively. The pump laser, sCMOS camera, and spectrometer were synchronized throughout the experiment to ensure that each spectrum and image corresponded to a single pump pulse. A three-dimensional motorized stage was employed for stage scanning and high-throughput measurements.

### Fabrication of microdroplet laser array

Rhodamine solution with a concentration of 10 mM was prepared by dissolving rhodamine B powder (Aladdin, No. 81-88-9) in deionized (DI) water. In order to reduce evaporation, 50% (v/v) glycerol (Aladdin, No. 56-81-5) was added into the rhodamine solution. The mixed solution was used as ink for printing.

The microdroplet laser array was fabricated on a glass slide. First, the glass slide was cleaned using a plasma cleaner (PLUTOVAC, PLUTO-T) for 1 min. The cleaned slide was immersed in a fluorosilane-modified silicon dioxide coating solution and incubated for 1 minute. After heating at 70 °C on a hot plate for 5 min, a superhydrophobic layer was formed on the glass slide. A commercial inkjet printer (EPSON, L130) was used for microdroplet generation.

### Actuating the microdroplets with ultrasound

A piezoelectric transducer attached on the glass slide was used to generate ultrasound. A sine wave with an amplitude of 3 V and a fixed frequency of 132.4003 kHz, generated by a function generator, was used to drive the piezoelectric transducer.

### Hyperspectral imaging

Hyperspectral images were recorded using the spectrometer. The laser signal was coupled into the spectrometer through the entrance slit and dispersed by a grating (600 lines.mm^−1^) according to wavelength, allowing different spectral components to be distinguished at various locations on the camera. The entrance slit was opened wide enough to collect the entire laser pattern.

### Calculation of relative correlation

We recorded the time-resolved laser emission spectra. Each spectrum can be written as a vector

1 $$A\left(t\right)=\left[{a}_{1}\left(t\right),\,{a}_{2}\left(t\right),\ldots ,\,{a}_{n}(t)\right]$$

with ${a}_{i}\left(t\right)$ denoting the intensity of *i*th data point in the laser spectrum. *n* is the number of data points in laser spectrum. The time-varying correlation can be calculated by using

2 $$r(t)=\frac{{\sum }_{i=1}^{n}({a}_{i}\left(t\right)-\overline{A\left(t\right)})({b}_{i}-\bar{B})}{\sqrt{({\sum }_{i=1}^{n}{({a}_{i}\left(t\right)-\overline{A\left(t\right)})}^{2})\cdot ({\sum }_{i=1}^{n}{({b}_{i}-\bar{B})}^{2})}}$$

Here, $\bar{A\left(t\right)}={\sum }_{i=1}^{n}{a}_{i}\left(t\right)/n$ is the average intensity of the laser spectrum. $B=\left[{b}_{1},\,{b}_{2},\ldots ,\,{b}_{n}\right]$ is the reference spectrum, i.e., the laser spectrum at $t=0$ and $\bar{B}$ is the corresponding average intensity. The relative correlation is defined as $R\left(t\right)=r\left(t\right)-{r}_{0}\left(t\right)$, with ${r}_{0}\left(t\right)$ denoting the moving average of *r*(*t*), which can be calculated as follows.

3 $${r}_{0}\left(t\right)=\frac{1}{k}\mathop{\sum }\limits_{j=1}^{k}r({t}_{j})$$

with *k* denoting moving window.

### SD calculation of relative correlation

The standard deviation of the temporal relative correlation can be given by

4 $$\sigma =\sqrt{\frac{1}{N}\mathop{\sum }\limits_{j=1}^{N}{(R({t}_{j})-\mu )}^{2}}$$

Here, *N* is the number of laser spectra, which corresponds to the number of data points in the relative correlation curve. $\mu =\frac{1}{N}(R\left({t}_{1}\right)+R\left({t}_{2}\right)+\ldots +R\left({t}_{N}\right))$ is the average value of relative correlation.

### Measuring the viscosity of BSA solution

The BSA stock solution with a concentration of 5 mM was prepared by dissolving BSA powder (Aladdin, No. B265994) in phosphate-buffered saline (PBS). For the measurements in Fig. 4d, BSA solutions with concentrations ranging from 0.75 to 2.25 mM were prepared by diluting the stock solution with PBS. For the measurements in Fig. 4e, NaCl solutions with concentrations varying from 0 to 600 mM were mixed with 3 mM BSA solution at a volume ratio of 1:1. For the measurements in Fig. 4f, 1.5 mM BSA solution with different pH values were prepared by adjusting the pH with acetate (Aladdin, No. A116166) and NaOH (Aladdin, No. S431790) solutions.

### Preparation of serum

This study was approved by Medical Ethics Committee of Chongqing General Hospital (approval no. KY S2025-012-01). Human blood samples were freshly collected in Chongqing General Hospital. Then, the samples were centrifuged at 4000 r.min^−1^ for 10 min. The resulting supernatant was transferred to a clean microcentrifuge tube and stored at −80 °C for future use. In scanning experiment, a 10 times dilution of serum with PBS was used. The diluted serum was mixed with rhodamine B solution (50 mM) and glycerol with a volume ratio of 18:1:1. The mixed solution was further injected into the printer for microdroplets fabrication.

### Analysis of the viscosity map

The viscosity map was obtained through stage scanning (More details about the scanning experiment can be found in Supplementary Fig. S16). The average viscosity was used to quantify the overall viscosity of the entire droplet.

## Supplementary information

SUPPLEMENTAL MATERIAL for Laser-Emission Vibrational Microscopy of Microdroplet Arrays for High-Throughput Screening of Hyperlipidemia

## Data Availability

All data are available within the Article and Supplementary Files, or available from the corresponding authors on reasonable request.
