# Supplementary material for: Laser-emission vibrational microscopy of microdroplet arrays for high-throughput screening of hyperlipidemia
Source: Light Sci Appl. 2025 Sep 17;14:327. doi: 10.1038/s41377-025-02015-5 (PMC12443976; doi:10.1038/s41377-025-02015-5)
Supplement: Supplementary file 1 — SUPPLEMENTAL MATERIAL for Laser-Emission Vibrational Microscopy of Microdroplet Arrays for High-Throughput Screening of Hyperlipidemia [file 41377_2025_2015_MOESM1_ESM.docx]

Supplementary Information for:

**Laser-Emission Vibrational Microscopy of Microdroplet Arrays for High-Throughput Screening of Hyperlipidemia**

Zhonghao Li^1^, Zhihan Cai^1^, Yuhan Wang^2^, Yuliang Liu^1^, Guifeng Li^1^, Xi Yang^3^, Ming Deng^1^, Yu-Cheng Chen^4,*^, Jichun Yang^2,*^, Yang Luo^2,*^, Chaoyang Gong^1,*^, Tao Zhu^1^

^1^ Key Laboratory of Optoelectronic Technology and Systems (Ministry of Education of China), School of Optoelectronic Engineering, Chongqing University, Chongqing 400044, China.

^2^ Department of Laboratory Medicine, Chongqing General Hospital, School of Medicine, Chongqing University, Chongqing 401147, China.

^3^ Key Laboratory of Optical Fiber Sensing and Communications (Ministry of Education of China), University of Electronic Science and Technology of China, Chengdu, Sichuan 611731, China.

^4^ School of Electrical and Electronic Engineering, Nanyang Technological University, Singapore 639798, Singapore

Correspondence Emails: [yucchen@ntu.edu.sg](mailto:yucchen@ntu.edu.sg); [yangjichun@cqu.edu.cn](mailto:yangjichun@cqu.edu.cn); [Luoy@cqu.edu.cn](mailto:Luoy@cqu.edu.cn); [cygong@cqu.edu.cn](mailto:cygong@cqu.edu.cn)

**CONTENTS**

[1. Experimental setup 3](#_Toc201132550)

[2. Characterization of microdroplet array 3](#_Toc201132551)

[3. Characterization of laser emission 4](#_Toc201132552)

[3.1 Q-factor evaluation 4](#_Toc201132553)

[3.2 Hyperspectral image of lasing microdroplet 4](#_Toc201132554)

[4. The deformation of microdroplet induced by ultrasound 5](#_Toc201132555)

[5. Extracting mechanical vibration with laser spectra 6](#_Toc201132556)

[5.1 Temporal evolution of Q-factor 6](#_Toc201132557)

[5.2 Temporal evolution of optical path 6](#_Toc201132558)

[5.3 Statistical distribution of relative correlation 7](#_Toc201132559)

[5.4 Frequency spectrum of temporal relative correlation 8](#_Toc201132560)

[5.5 Under sampling effect 8](#_Toc201132561)

[5.6 Mechanical vibrations of microdroplets under different driving voltages 10](#_Toc201132562)

[5.7 Mechanical vibrations of microdroplets with various sizes 11](#_Toc201132563)

[6. Viscosity of glycerol solution 11](#_Toc201132564)

[7. Measuring viscosity with SD 12](#_Toc201132565)

[8. Stage scanning 13](#_Toc201132566)

[8.1 Reconstructing of viscosity map 13](#_Toc201132567)

[8.2 Influence of sample size in SD calculation 13](#_Toc201132568)

[8.3 Influence of spot size on SD calculation 14](#_Toc201132569)

[8.4 Comparison of stage scanning and fixed measurement 15](#_Toc201132570)

[8.5 Stability of microdroplet size during scanning 16](#_Toc201132571)

[9. Blood samples 17](#_Toc201132572)

[10. Feasibility of clinic applications 18](#_Toc201132573)

# 1. Experimental setup

The experimental setup was illustrated in Fig. S1. An upright microscope integrated with the three-dimensional motorized stage was used for the laser excitation and signal collection (Fig. S1a). The spectrometer mounted with two cameras was used for spectral images (camera 1) and time-resolved laser spectra (camera 2) collection, respectively (Fig. S1b). The mounting method of the glass slide on the microscope stage was illustrated in the Fig. S1c.

**
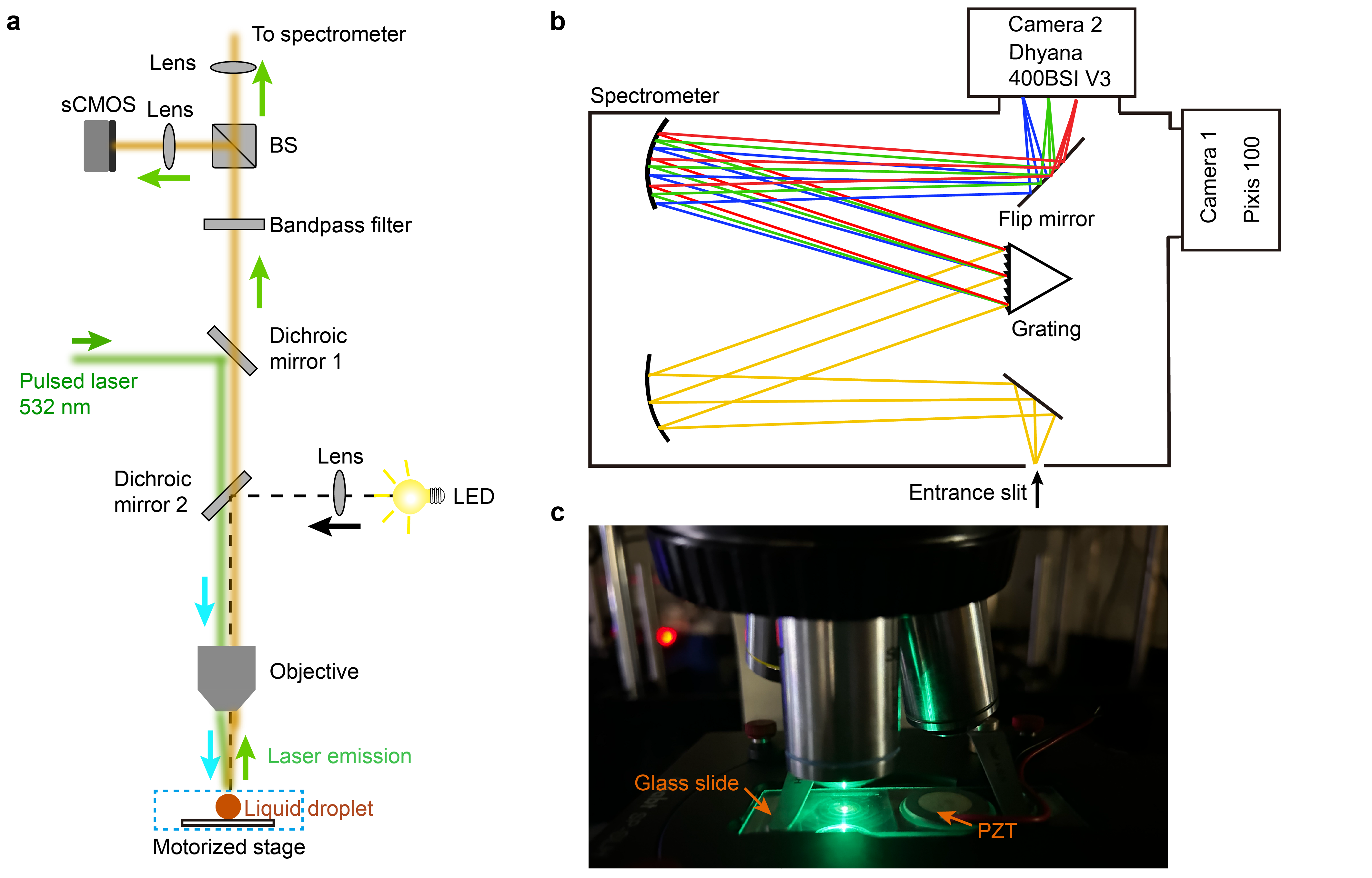
**

**Figure S1.** (**a**, **b**) Illustration of the upright microscopy (**a**) and the spectrometer (**b**). (**c**) Glass slide on microscope stage.

# 2. Characterization of microdroplet array

We modified the Epson L130 printer by replacing the ink cartridge with centrifuge microtubes, which were filled with sample solutions for printing (Fig. S2a). The printer parameters such as voltage, pulse frequency, nozzle diameter of the printer are set by the manufacturer and cannot be modified. Each travel cycle of the printhead ejects a droplet with an approximate volume of ~ 4.2 pL, corresponding to a droplet diameter of ~20 μm. The size of the droplet can be controlled by increasing travel cycles of printhead. The microdroplet array was fabricated on a superhydrophobic surface (Fig. S2b), and the program controls the location. Due to the precise control of droplet size by the inkjet printer, the droplets are uniform sized (~ 20 μm) with a variation of 2.3 % (Fig. S2c). The microdroplets exhibit an average contact angle of approximately 133.5°, forming a nearly spherical morphology for lasing (Fig. S2d).

**
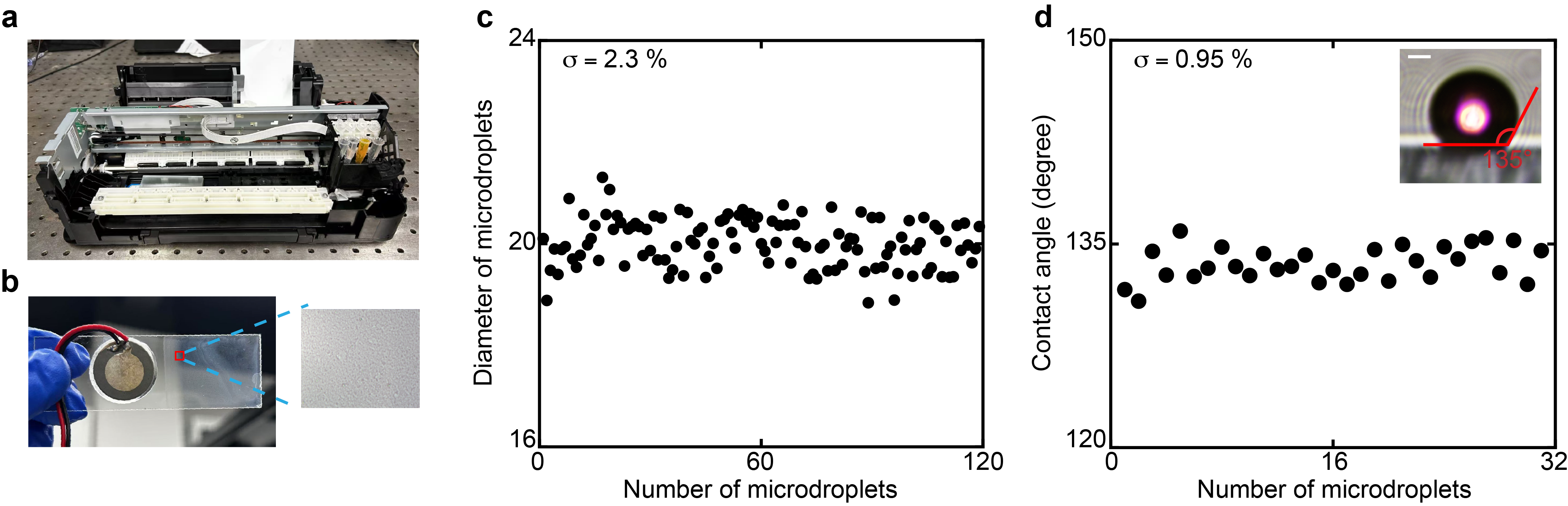
**

**Figure S2. Characterization of microdroplet array**. (**a**) Modified commercial Epson L130 printer. (**b**) Super-hydrophobic surface on glass slide. Inset, enlargement of the region in the red box. (**c**, **d**) Characterization of the diameters (**c**) and contact angles (**d**).

# 3. Characterization of laser emission

## 3.1 Q-factor evaluation

The threshold condition for a four-level laser system can be expressed as^1^

$$\eta D_{1}\sigma_{e}\left( \lambda\right)=\eta{\sigma_{a}\left( \lambda\right)D}_{0}+\frac{2\pi n}{\lambda_{L}Q} \text{(S1)}$$

Here, $\eta$ is the fraction of energy that interacts with the gain molecules. In a microdroplet laser, the optical field is mostly confined within the droplet, hence $\eta=1$ is employed as an approximation. $D_{0}$ and $D_{1}$ are the density of dye molecules in the ground state and the lowest excited singlet state, respectively. $\sigma_{e}\cong4\times{10}^{-16}$ cm^2^ and $\sigma_{a}\cong1\times{10}^{-17}$ cm^2^ represent the emission and absorption cross-sections of rhodamine B, respectively^2^. $\lambda_{L}$ denotes the laser wavelength and *Q*stands for the Q-factor. $n$ is the effective refractive index of the WGMs. Thus, the fraction of molecules in the excited state can be given by

$$\gamma=\frac{D_{1}}{D_{0}+D_{1}}=\frac{\sigma_{a}\left( \lambda\right)}{\sigma_{e}\left( \lambda\right)}(1+\frac{2\pi n}{\lambda_{L}D\eta Q\sigma_{a}(\lambda)}) \text{(S2)}$$

Here, $D=D_{0}+D_{1}$ denotes the density of total gain molecules. According to the rate equation for a four-level laser system, $\gamma$ can also be approximated as^3^

$$\gamma=\frac{\omega_{p}\tau_{rad}}{1+\omega_{p}\tau_{rad}} \text{(S3)}$$

Here, $\omega_{p}=\sigma_{a}/(E_{0}\Delta t)$ is the normalized pump intensity. $I_{th}$ is the laser threshold. $E_{0}=hc/\lambda_{p}$ is the photon energy of the pump. $\Delta t$ is the pulse width of the pump laser. $\tau_{rad}=$ 2.3 ns is the lifetime of rhodamine B in the excited state^4^. Given the threshold excitation intensity of 10.5 μJ mm-2, $\gamma$ can be calculated to be 0.0183. By letting γ, the Q-factor is estimated to be around 6$\times$10^4^.

## 3.2 Hyperspectral image of lasing microdroplet

The laser pattern was dispersed by a diffractive grating in spectrometer according to its wavelength. The complete spectral image of the microdroplet in Fig. 2a was illustrated in Fig. S3.

**
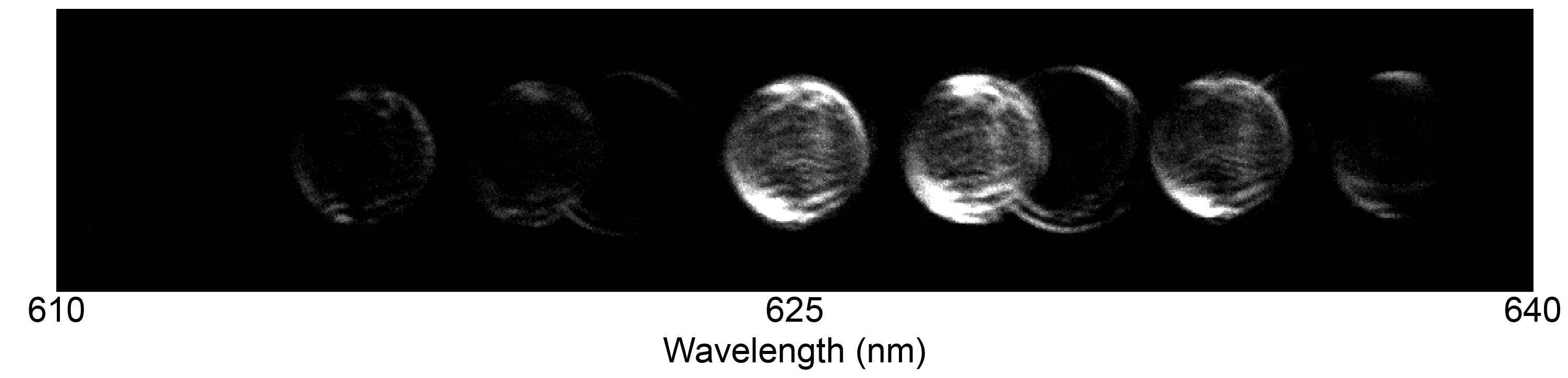
**

**Figure S3.** Spectral image of a lasing microdroplet.

**3.3 Stability of laser emission**

We investigated the laser spectra of microdroplets with varying sizes. As illustrated in Fig. S4, laser emission from larger droplets shows strong mode competition, which we think will deteriorate the sensing performance. In order to achieve a better laser stability, the droplet size was kept at 20 µm throughout the experiment, which is the smallest size the printer can fabricate.


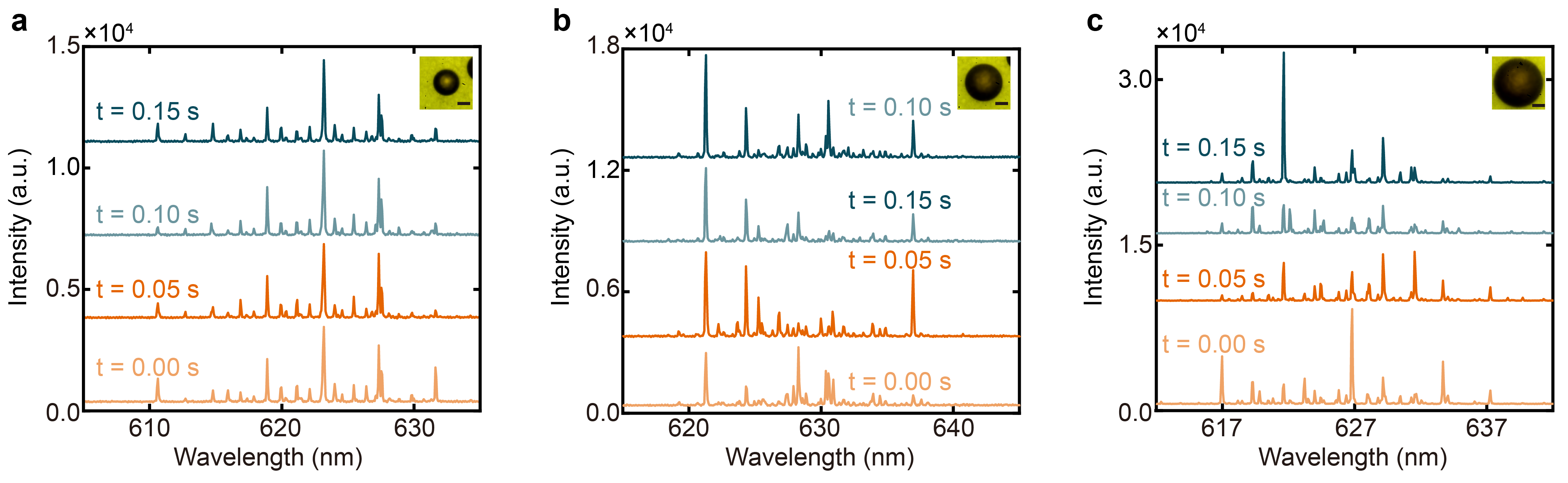


**Figure S4. The mode competition in spectrum of microdroplets with varying diameters**. (**a**, **b**, **c**) The spectra of microdroplets with diameter of 20 µm (**a**), 30 µm (**b**), 40 µm (**c**). Inset, image of the microdroplet with diameter of 20, 30 and 40 µm. Scale bar: 10 µm.

# 4. The deformation of microdroplet induced by ultrasound

Natural frequency of the microdroplet can be expressed as^5^

$$f_{0}=\frac{1}{2\pi R}\sqrt{\frac{\sigma}{\rho R}} \text{ (S4)}$$

Here, *R =* 10 μm is the radius of microdroplet, $\sigma$ and $\rho$ are surface tension and density of microdroplet, respectively. The natural frequency is estimated to be around 1.3$\times$10^5^ Hz, which is close to the driving frequency (1.324$\times$10^5^ Hz) used in our experiment. The radiation force applied by ultrasound can be given by^6^

$$F=AP_{0}\cos\left( \omega t+\varphi\right) \text{(S5)}$$

where, *A* is the pressure’s action area. *P_0_* and $\varphi$ are sound pressure and phase, respectively. $\omega$ is driving angular frequency. The radiation force induces periodic compression and expansion of microdroplet. The degree of deformation is defined by^7^

$$D=\frac{R-\Delta R}{R+\Delta R}\propto\frac{FR}{\sigma A}\cdot\frac{1}{1+\left( \omega\tau\right)^{2}} \text{(S6)}$$

Here, $\Delta R$is the morphology deformation $\tau=\eta R/\sigma$ is the relaxation time of microdroplet. $\eta$ is the viscosity coefficient. The morphological changes in microdroplet alter the periodic switching of WGMs oscillation direction, resulting in a significant wavelength shift and intensity fluctuation of laser peaks.

#

# 5. Extracting mechanical vibration with laser spectra

## 5.1 Temporal evolution of Q-factor

According to the laser theory, the laser intensity can be written as^3^

$$I_{laser}=A(\frac{I_{pump}}{I_{th}}-1) \text{(S7)}$$

Here, $I_{pump}$ is the pump intensity. *A* is a constant. Combining with Eqs. S3 and S7, we derived that

$$I_{laser}=A\left[ \frac{I_{pump}\sigma_{a}\tau_{rad}}{E_{0}\Delta t}\left( \frac{1}{\gamma}-1 \right)-1 \right] \text{(S8)}$$

This equation shows the dependence of the laser intensity on the fraction of molecules in the excited state$\gamma$. According to the analysis above, the Q-factor’s temporal evolution of the TE_143_ laser mode can be calculated by the intensity fluctuations given in Fig. 3c. As shown in Fig. S5, the Q-factor responds to ultrasound stimulus with periodic fluctuations.


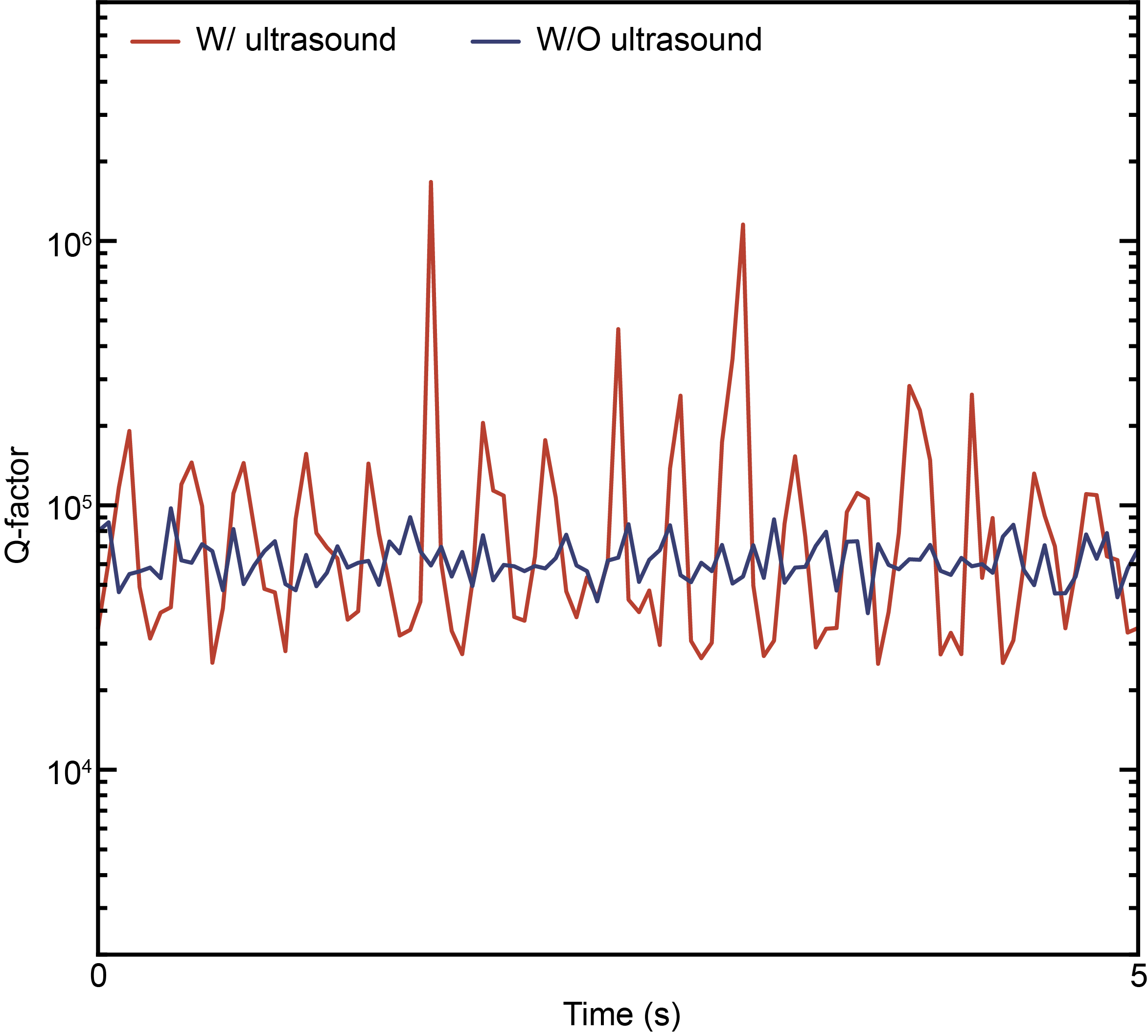


**Figure S5.** Reconstructed temporal evolution of Q-factor.

## 5.2 Temporal evolution of optical path

The resonance condition for the WGM can be expressed as^8^

$\text{2}\text{π}n_{eff}R=m\lambda_{m}$ (S9)

Here, $n_{eff}$ is the effective refractive index of the mode, *R* is the radius of microdroplet, *m* is azimuthal quantum number, $\lambda_{m}$ is resonant wavelength. The ultrasound applied to the microdroplet induces mechanical vibrations, resulting in periodic wavelength shifts of laser peaks. Thus, the optical path can be given by

$$\Delta R=\frac{m\Delta\lambda}{{2\pi n}_{eff}} \text{(S10)}$$

$\Delta\lambda$ is the wavelength shift induced by morphology deformation $\Delta R$. The optical path can be estimated by using *P* $={2\pi n}_{eff}(R+\Delta R)$. We calculate the time-varying optical path in Fig. S6, a maximum optical path change is about 6.5 nm.

**
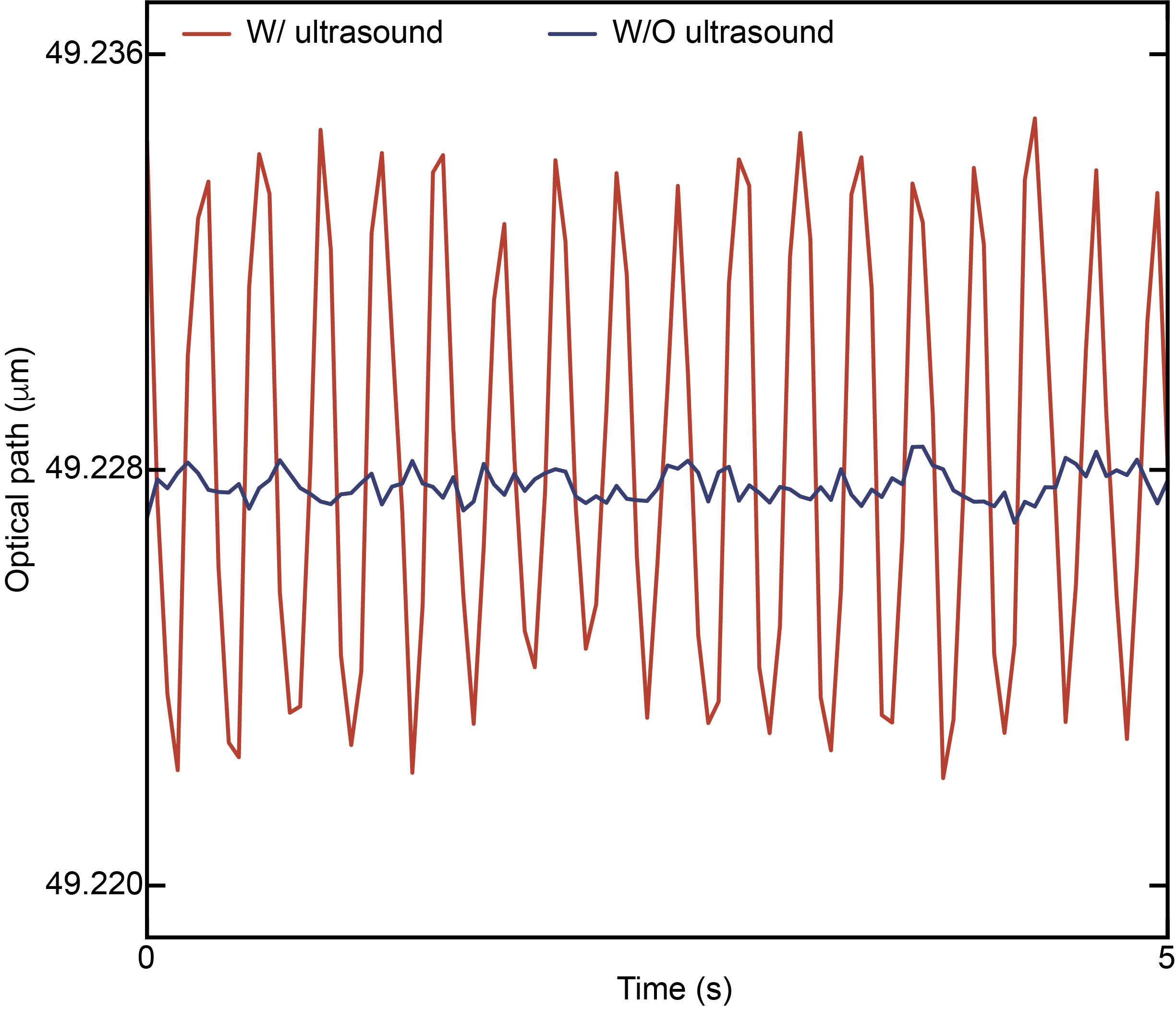
**

**Figure S6.** Temporal evolution of optical path.

## 5.3 Statistical distribution of relative correlation

As shown in Fig. S7a, the temporal relative correlation curve was calculated by subtracting the smooth curve from the temporal correlation curve. The statistical distribution of the relative correlation follows a normal distribution (Fig. S7b).


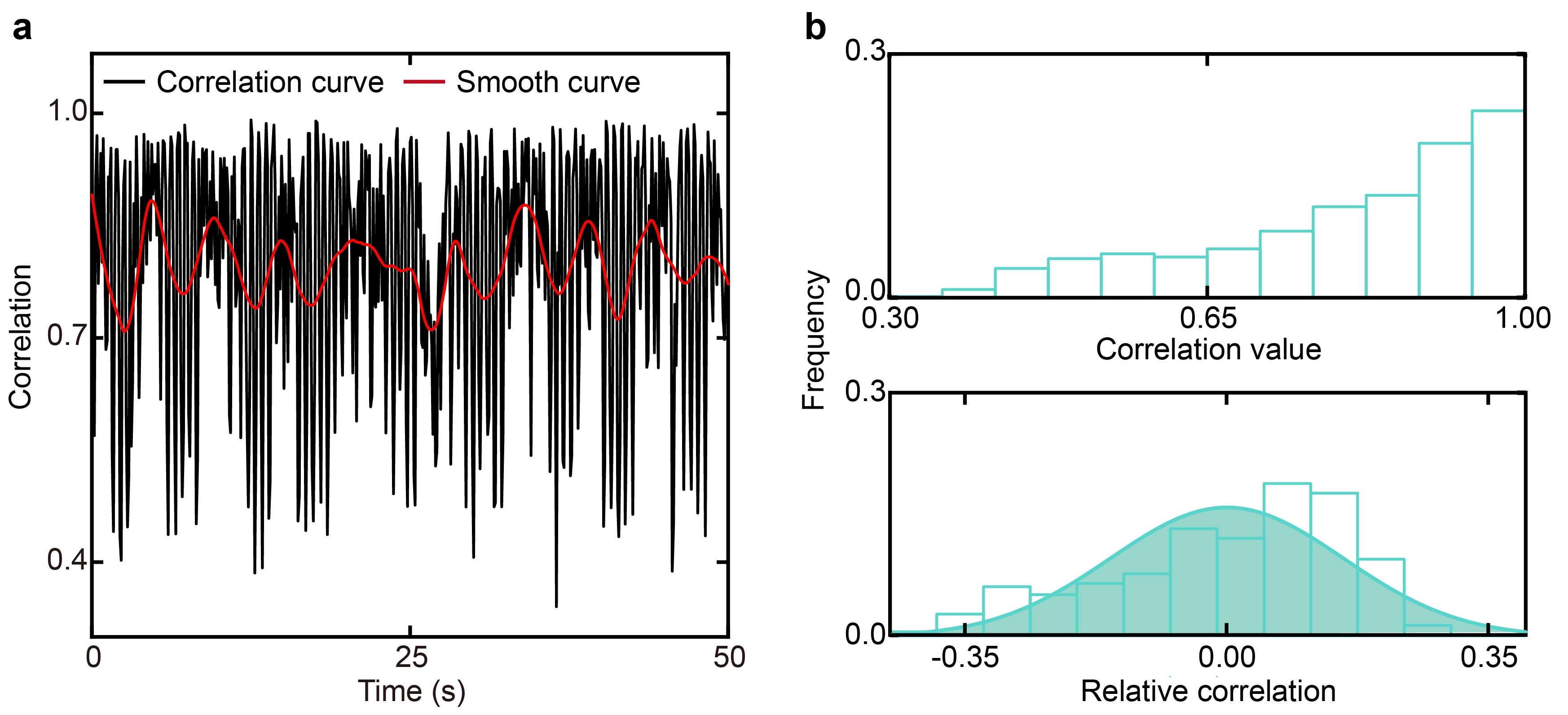


**Figure S7.** The statistical distribution of temporal relative correlation. (**a**) The temporal correlation curve (**black**) of laser spectra. The smooth curve (**red**) was obtained using a 40-point neighbor averaging. (**b**) The statistical distribution of correlation (**top**) and relative correlation (**bottom**).

## 5.4 Frequency spectrum of temporal relative correlation

The fast Fourier transform (FFT) spectrum of the temporal relative correlation curve in Fig. 3f is shown in Fig. S8a, which has a sharp peak at $f_{1}=$ 0.3 Hz. Similar result was observed in the FFT spectrum of wavelength shifts (Fig. S8b) and intensity fluctuations, respectively (Fig. S8c). This result indicates that the temporal relative correlation curve accurately captures the features of the laser spectra.

**
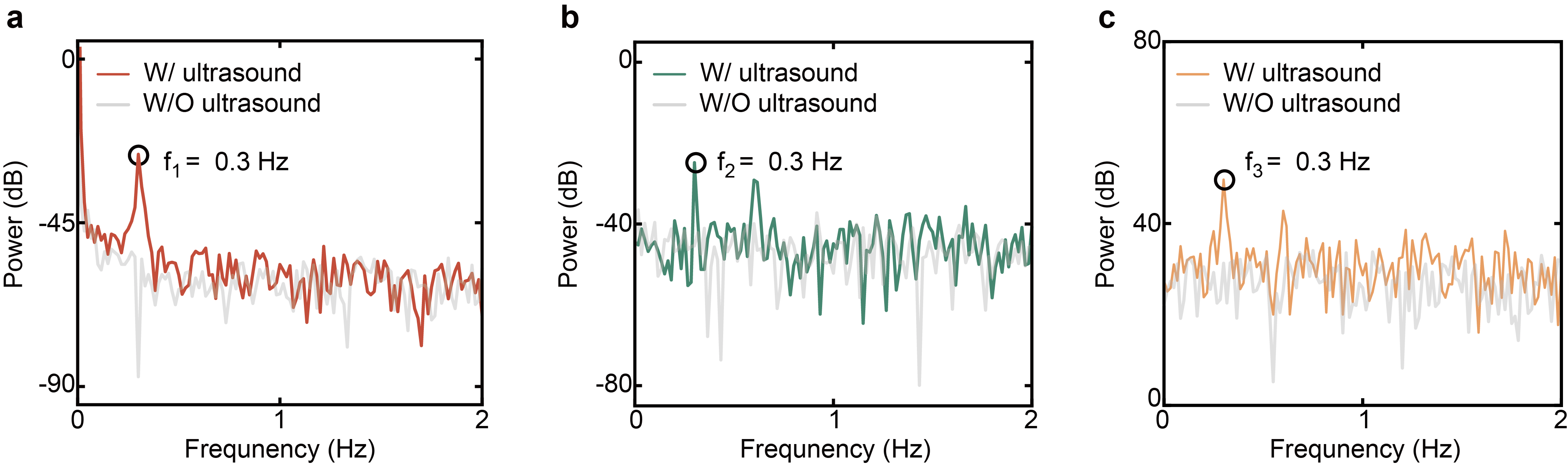
**

**Figure S8.** FFT spectrum of temporal relative correlation (**a**) wavelength shifts (**b**) and intensity fluctuations (**c**).

## 5.5 Under sampling effect

Figure S11a shows the schematic illustration of the under sampling effect. The pulsed laser samples the continuous waveform of ultrasound at a low repetition rate, producing a sequence of discrete values that constitute a lower-frequency waveform. The continuous ultrasound signal applied to the microdroplet can be expressed as

$x\left( t \right)=Acos(2\pi f_{0}t+\varphi)$ (S11)

Here, *A*, $f_{0}$ and $\varphi$ are amplitude, frequency, and initial phase, respectively. The continuous ultrasound signal was sampled with a sampling period of $T_{s}=1/f_{s}$. Here, $f_{s}$ is sampling frequency (pump repetition rate). The sampling signal can be given by

$$x\left( t \right)=\sum_{k=-\infty}^{\infty} \cos\left( 2\pi\frac{f_{0}}{f_{s}}+\varphi\right)\delta\left( t-kT_{s} \right) \text{(S12)}$$

Here, *k* is an integer. Under sampling effect occurs when the Nyquist sampling theorem is not satisfied ($f_{s}<2f_{0}$). $f_{0}$ was mapped to the first Nyquist region ($-f_{N}$ to $f_{N}$). Here, $f_{N}=f_{s}/2$ is Nyquist frequency. The frequency after the appearance of the under sampling effect can be given by^9^

$f=\left| f_{0}-nf_{s} \right|$ (S13)

with $n=\left\lfloor f_{0}/f_{s} \right\rfloor$ denoting the floor of $f_{0}/f_{s}$, which is an integer.

Fig. S9 shows the FFT spectra of the under sampling signals with various repetition rates. The frequencies of the obtained waveforms are dependent on the repetition rate of the pulsed laser. The results were consistent with the calculation in Eq. S13.

**
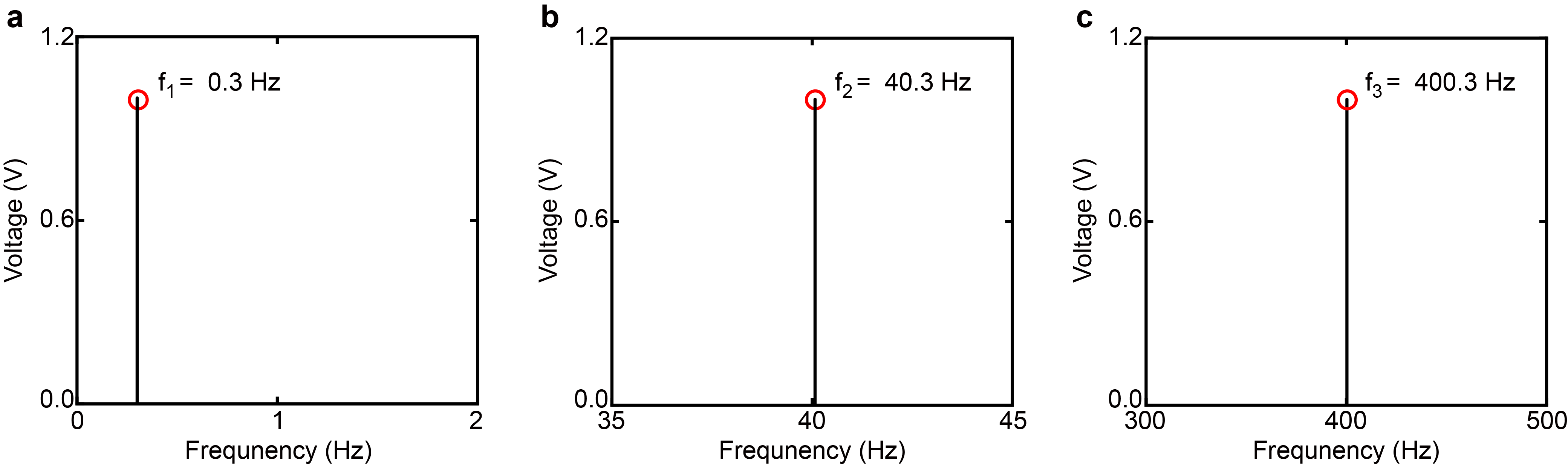
**

**Figure S9. Theoretical simulation of under sampling effect.** (**a**, **b**, **c**) FFT spectra of sampling signals with an under sampling frequency of 20 Hz (**a**), 120 Hz (**b**) and 1 kHz (**c**).

We also carried out experiment to demonstrate the accuracy of the theoretical calculation. As illustrated in Figs. S10a to S10c, we collected the temporal lasing spectra at the different pump repetition rates. After calculating the relative correlation as illustrated in Eqs. 2 to 3, we compared the FFT spectra in Figs. S10d to S10f. The results were consistent with the calculation in Fig. S9.


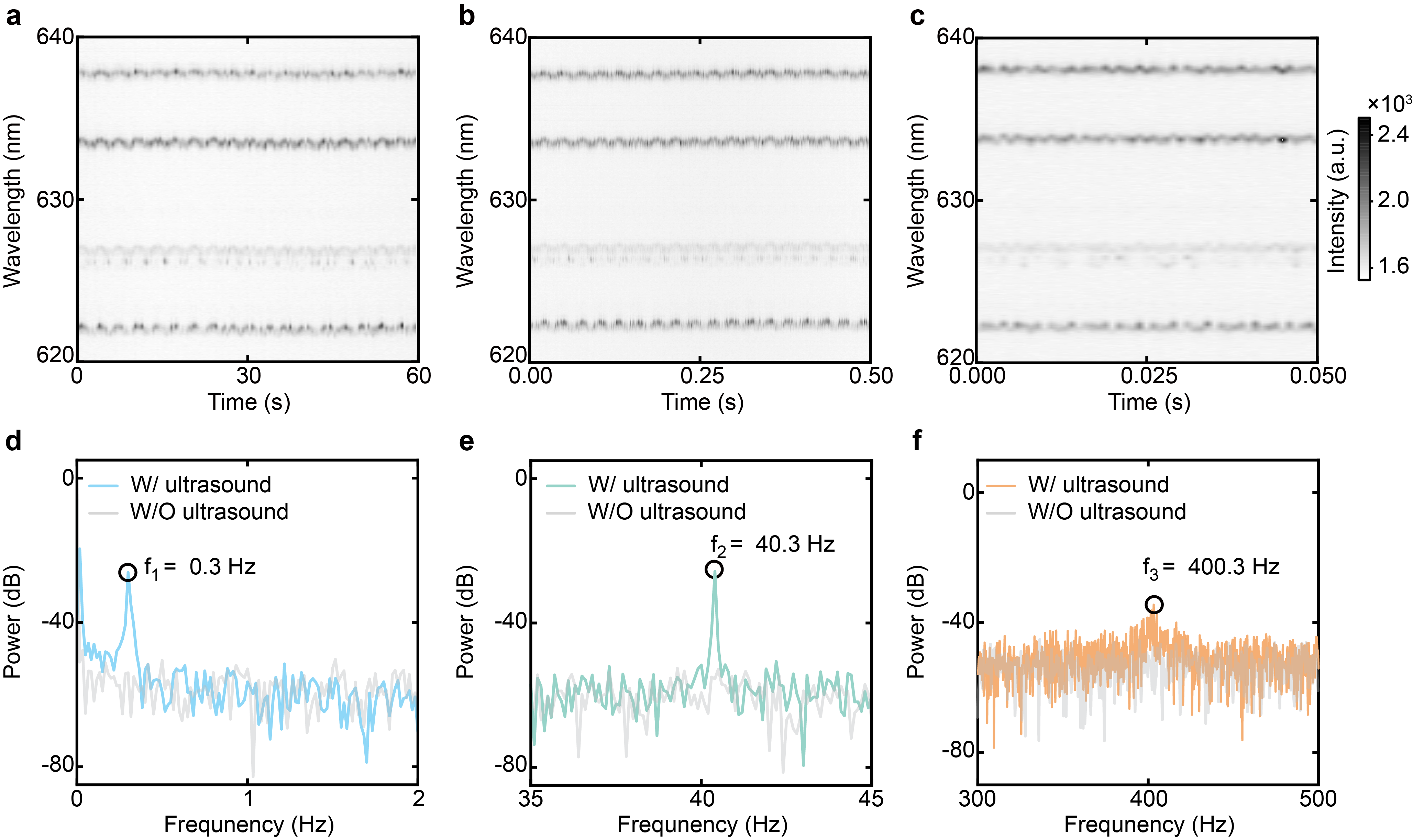


**Figure S10.** Temporal lasing spectra were obtained at pump repetition rate of 20 Hz (**a**), 120 Hz (**b**) and 1 kHz (**c**). The FFT spectra of temporal relative correlation at pump repetition rate of 20 Hz (**d**), 120 Hz (**e**) and 1 kHz (**f**).

We also carried out theoretical simulation to further illustrate the under sampling effect. As illustrated in Figs. S11b to S11e, the high-frequency ultrasound signal (132.4003 kHz) was sampled using pulsed lasers with various repetition rates, resulting in different lower-frequency waveforms. Although the under sampling effect distorts the signal, the standard deviation (SD) of the under sampling signal remains consistent with that of the original signal, which was used as the sensing output throughout the experiment.

**
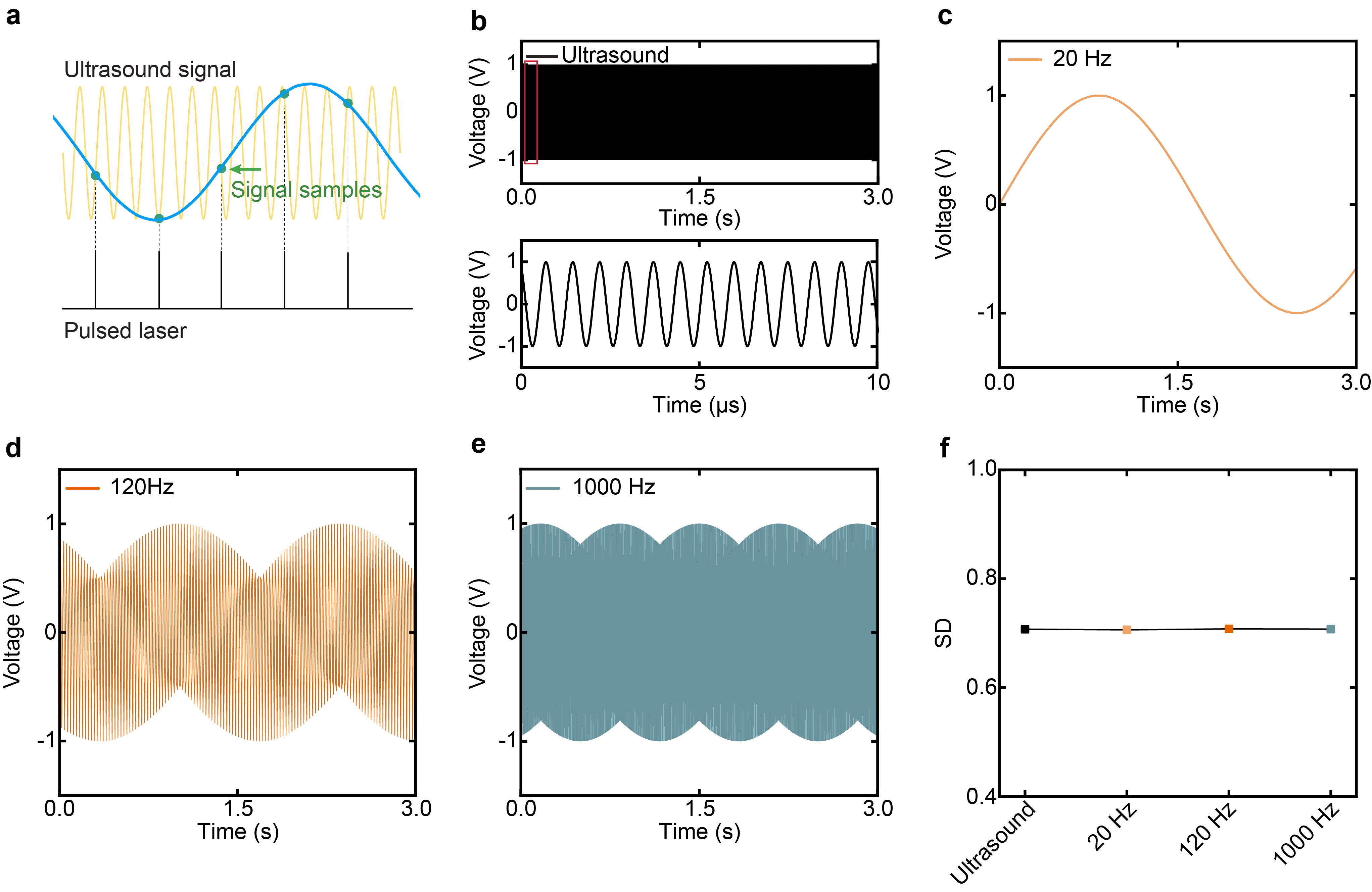
**

**Figure S11. Theoretical simulation of under sampling effect.** (**a**) The mechanism diagram of under sampling effect. (**b**) Simulation of ultrasound signal at 132.4003 kHz (**top**). Ultrasound signal within the red box (**bottom**) time window. (**c**, **d**, **e**) The sampling signals obtained at sampling frequency of 20 Hz (**c)**, 120 Hz (**d)**, 1 kHz (**e)**. (**f)** The SD of ultrasound signal and sampling signals.

## 5.6 Mechanical vibrations of microdroplets under different driving voltages

A piezoelectric transducer (PZT) attached on the glass slide (Fig. S2b) was used to generate ultrasound. As shown in Fig. S12a, the temporal relative correlation under different driving voltages of PZT. When the driving voltage is below 2 V, the SD of relative correlation is nearly zero, indicating that the microdroplet exhibits no vibration (Fig. S12b). Considering the measurement sensitivity and viscosity coefficient of the serum sample, we selected a driving voltage of 3 V for the experiment.

**
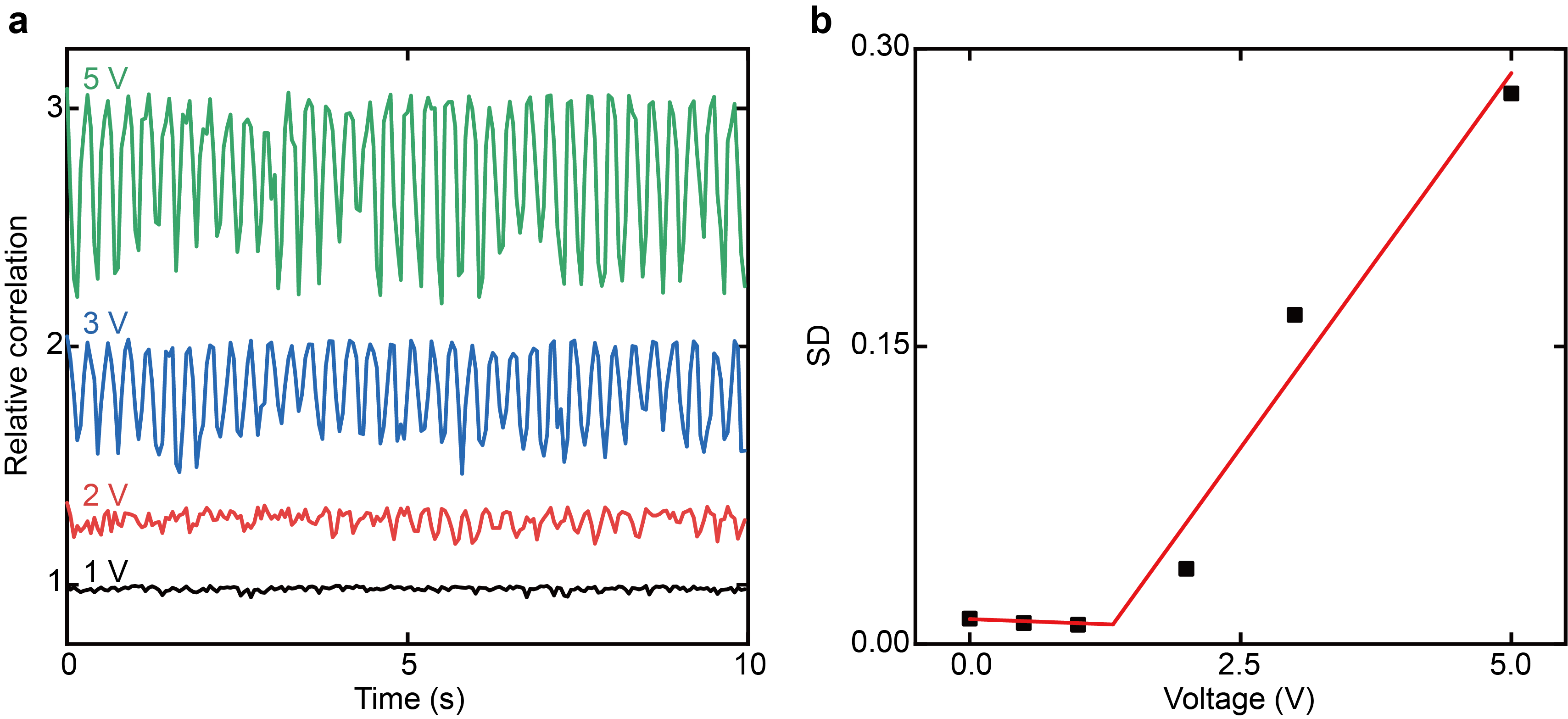
**

**Figure S12.** (**a**) The time-varying relative correlation under different driving voltages. (**b**) The SD of temporal relative correlation.

## 5.7 Mechanical vibrations of microdroplets with various sizes

We fabricated microdroplets of various sizes and tested their response to ultrasound. As shown in Fig. S13, the SD of temporal relative correlation decreases with an increasing droplet diameter. This phenomenon is due to the greater inertia of larger droplets, making them more resistant to vibration.

**
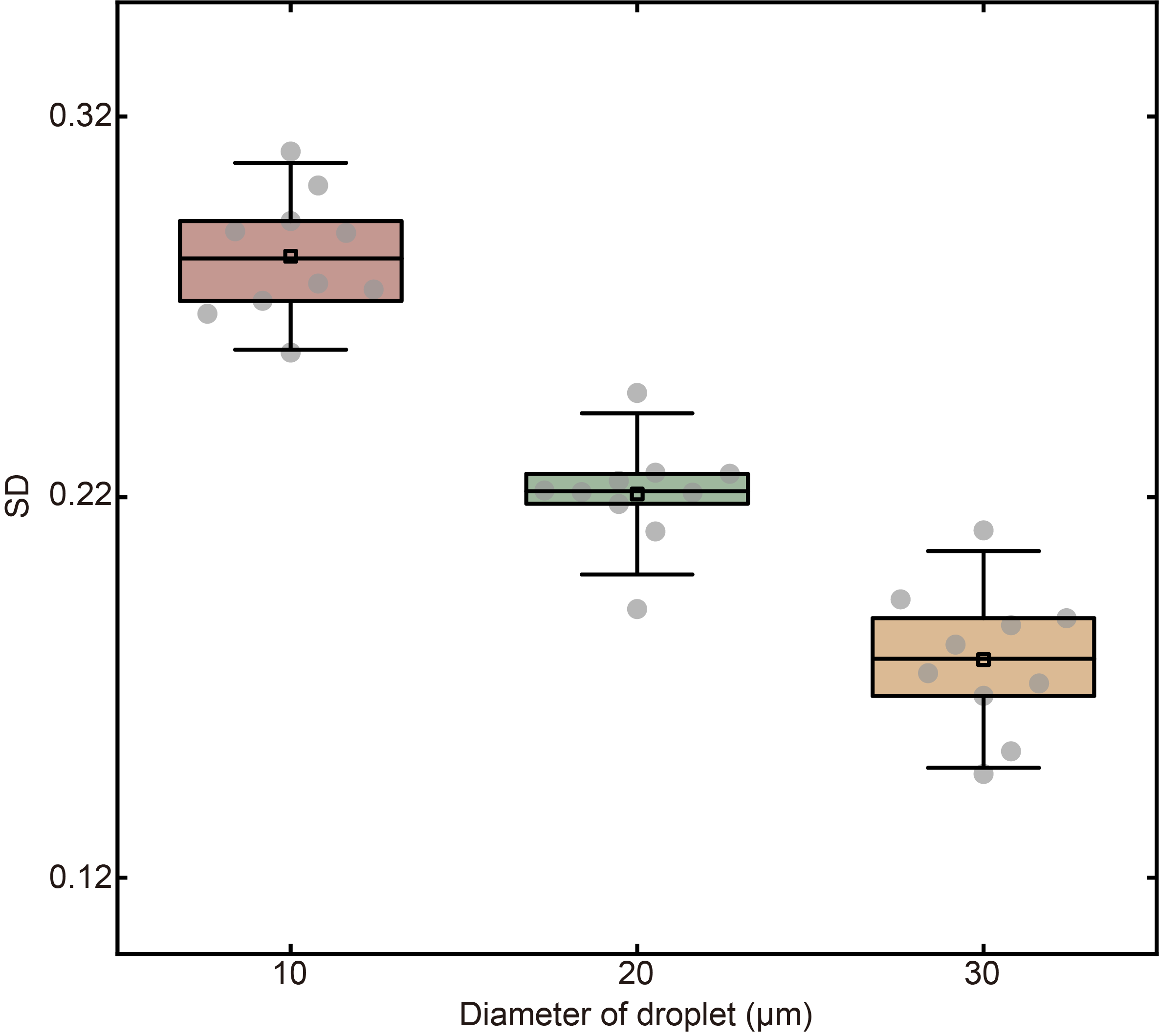
**

**Figure S13.** The SD of the temporal relative correlation of microdroplets with different diameters. Error bars represent standard deviations of 10 measurements.

# 6. Viscosity of glycerol solution

The relationship between viscosity and glycerol concentration was given in Fig. S14^10^. This curve was used for the viscosity calibrating experiment in Fig. 4c.

**
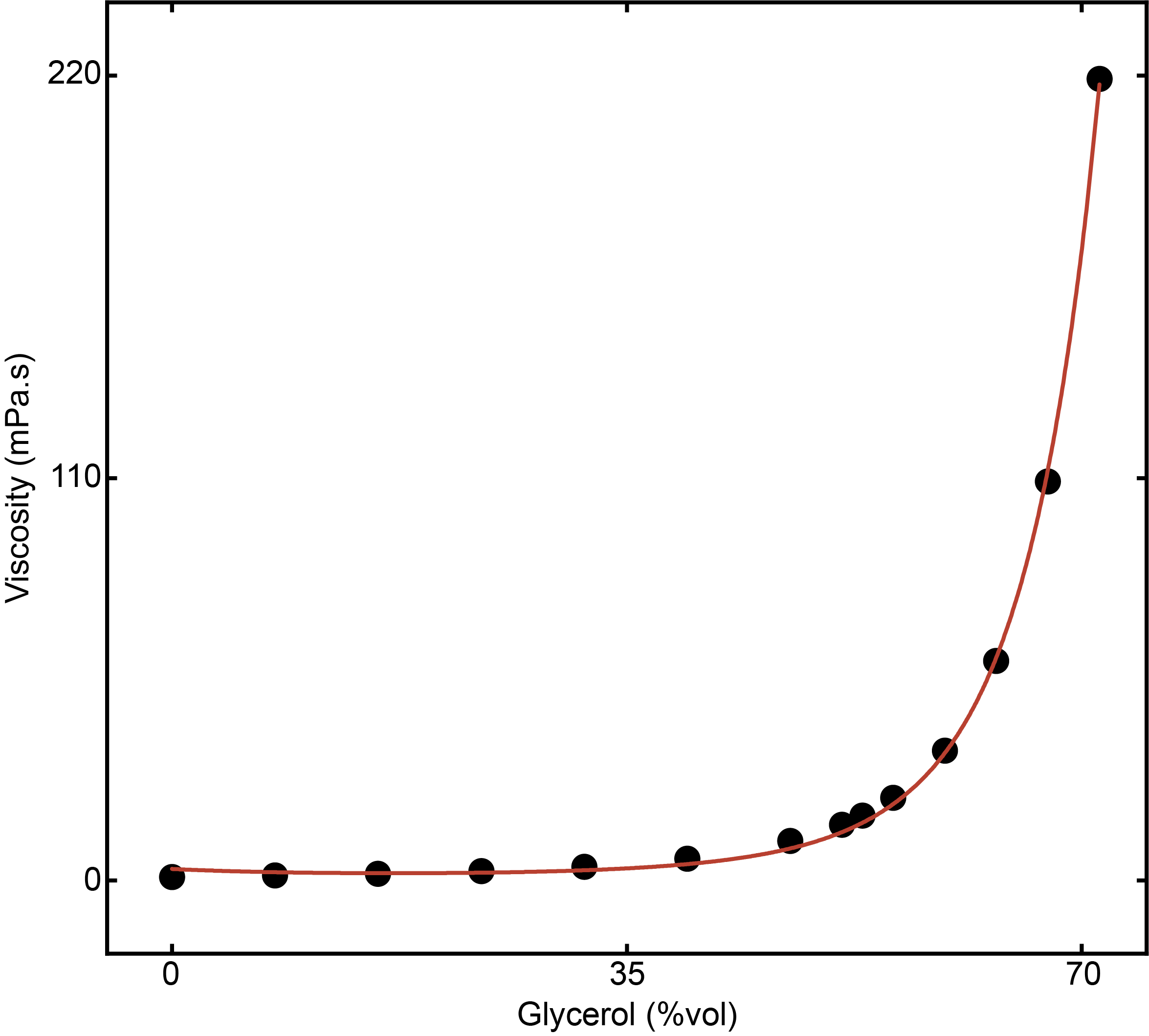
**

**Figure S14.** Viscosity of different concentrations of glycerol. All data are extracted from reference [10].

# 7. Measuring viscosity with SD

In our experiment, the SD of relative correlation curve was employed as the sensing output for viscosity calibration (Fig. 4c). In order to demonstrate the SD is connected to the absolute value of viscosity, we tested three types of solution, including bovine serum albumin (BSA), tween 20, dimethyl sulfoxide (DMSO) solution. The maximum relative error between the measured mean viscosity and the ground truth (Table S1) is 8.57 % in the DMSO solution (Fig. S15).


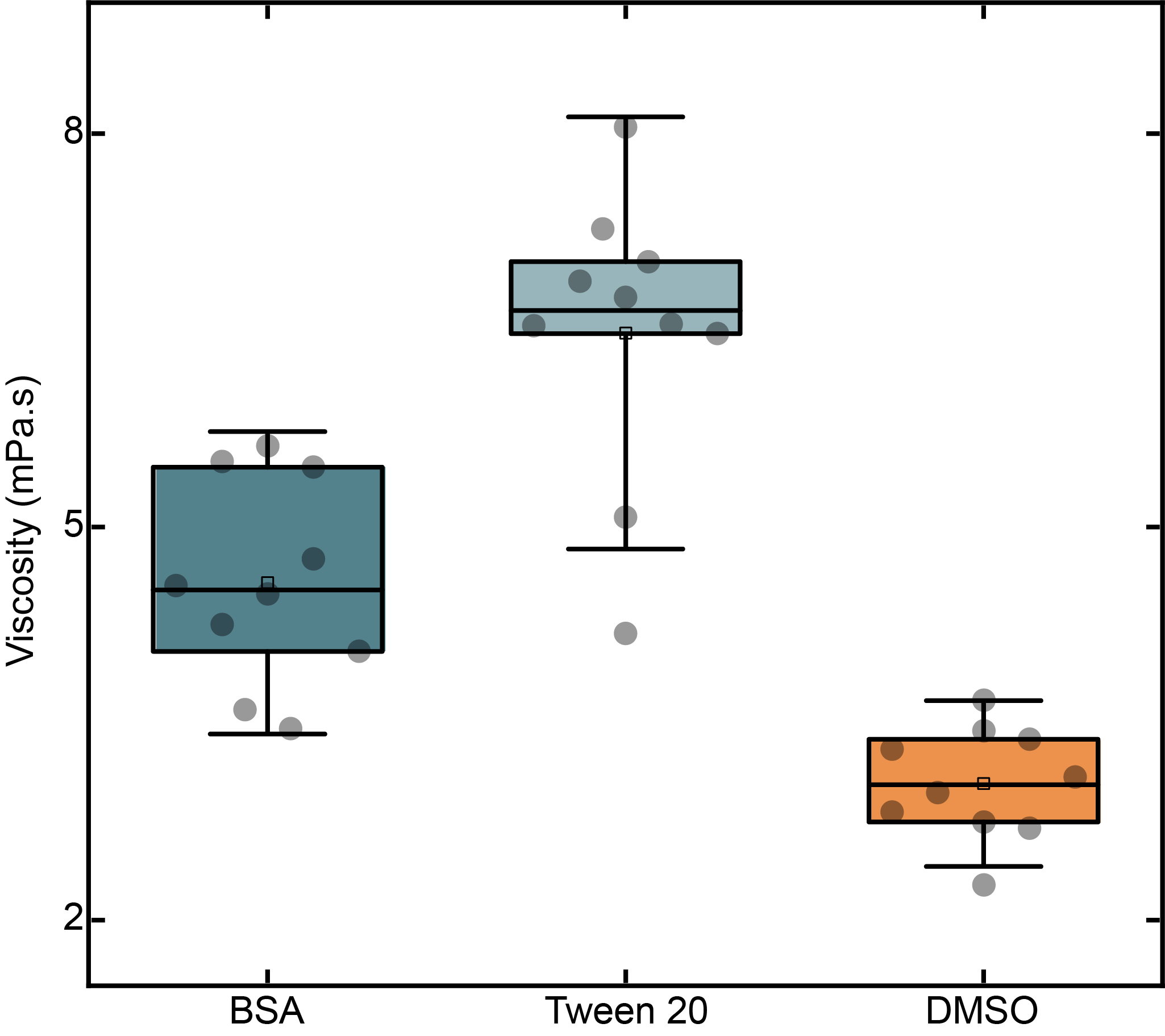


**Figure S15.** The measured viscosity of different solution. The error bars represent standard deviations of 10 viscosity measurements.

**Table S1** The measured viscosity and ground truth viscosity of the different solutions

| Type of solution | Measured viscosity  (mPa.s) | Ground truth  (mPa.s) | Division |
| --- | --- | --- | --- |
| BSA | 4.57 | 4.5^11^ | 1.75 % |
| Tween 20 | 6.48 | 7^12^ | 7.43 % |
| DMSO | 3.04 | 2.8^13^ | 8.57 % |

# 8. Stage scanning

## 8.1 Reconstructing of viscosity map

In the scanning experiment, the relative position of the pump spot on the droplet continuously changed over time (Fig. S16a). The laser spectra as a function of time during scanning was illustrated in Fig. S16b, where a discontinuous temporal signal was observed. We defined a rectangular scanning area, and no laser emission was observed when the pump laser is located outside the droplet. The temporal spectra were therefore divided into distinct regions (1, 2, 3, …) based on the presence or absence of lasing signal. In each region, the relative correlation was calculated using Eqs. (1) to (3), with the first spectrum of each region used as the corresponding reference. As illustrated in Fig. S16c, the resulting time-varying correlation curve reflects the spectral variations across different spatial locations on the droplet. Finally, using the calibration curve in Fig. 4c, the one-dimensional relative correlation curve was converted into a viscosity map based on the scanning time and speed.


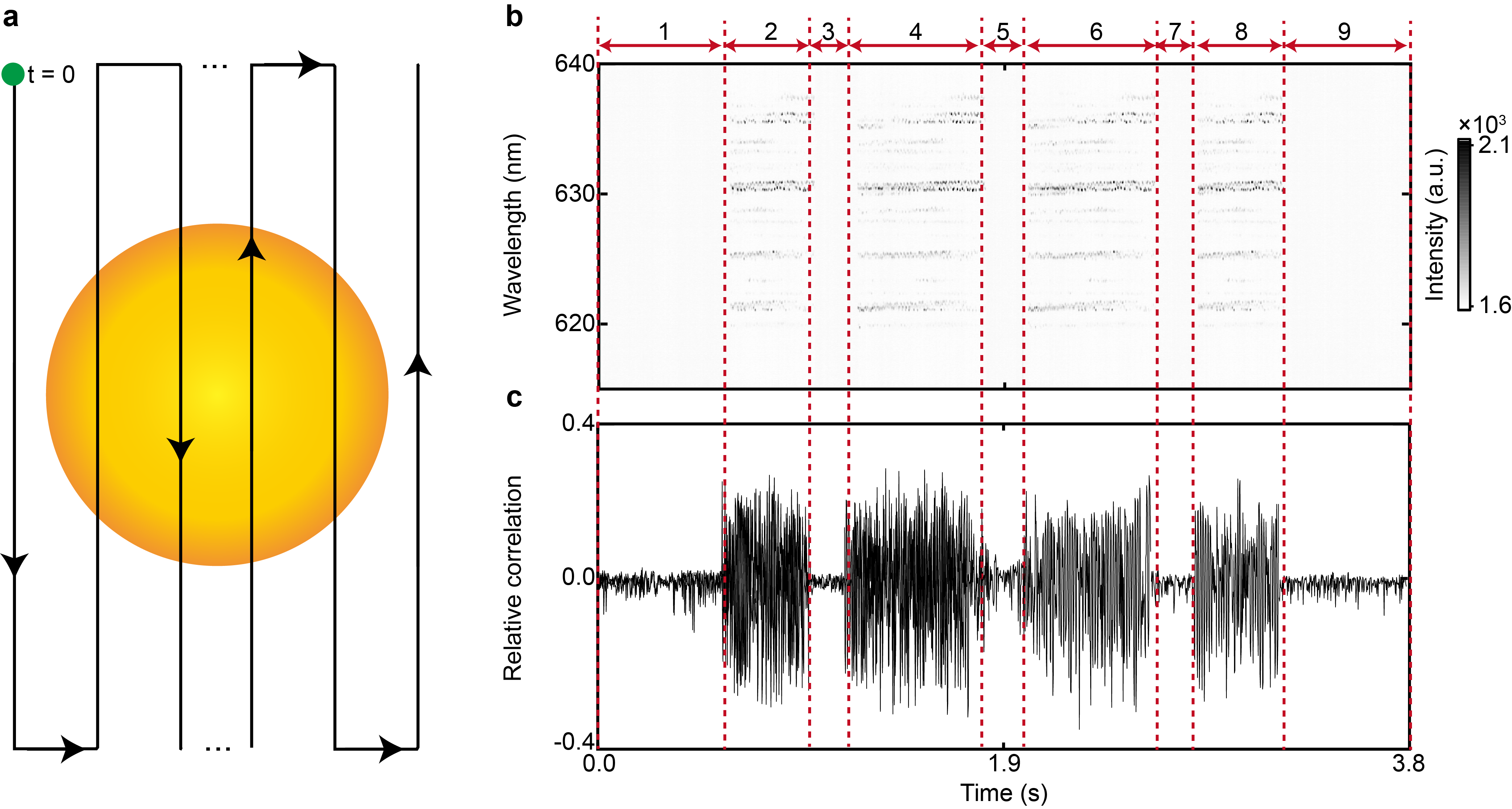


**Figure S16.** (**a**) Illustration of scanning measurement. (**b**, **c**) Temporal spectra (**b**) and the corresponding relative correlation curve (**c**).

## 8.2 Influence of sample size in SD calculation

We compared the scanning viscosity pattern with different sample size (N = 20, 100, 500) in Fig. S17. The uniformity of viscosity can be significantly improved by increasing the sample size in SD calculation. The mean values of the viscosity pattern with varying sample size exhibits a fluctuation of approximately 1.03 % (Fig. S17d).


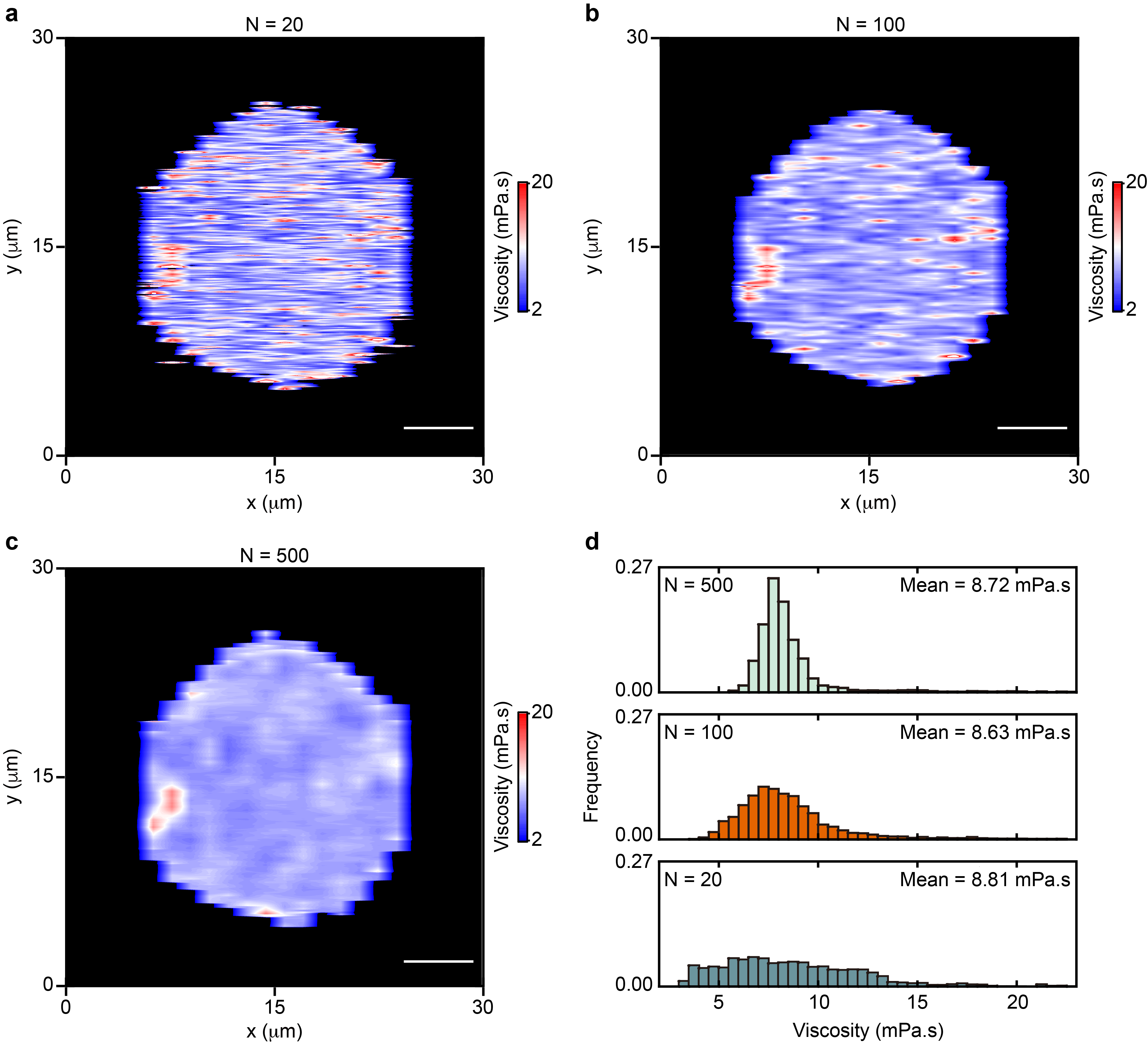


**Figure S17. (a**, **b**, **c**) The sample size for calculating the viscosity pattern was 20 (**a**), 100 (**b**) and 500 (**c**). (**d**) Statistical distribution of viscosity pattern with varying sample size. Scale bar: 5 µm.

## 8.3 Influence of spot size on SD calculation

The pump laser spot size in Figs. 3 and 4 is approximately 4.0 μm and is focused on the center of the droplet. In the scanning experiments shown in Figs. 5 and 6, the pump spot has a diameter of about 0.8 μm and scans across the microdroplet. The spot size is smaller than the typical slit width (~10 μm). As shown in Figs. S18, we demonstrated that the pump spot size has negligible influence on the viscosity measurement.


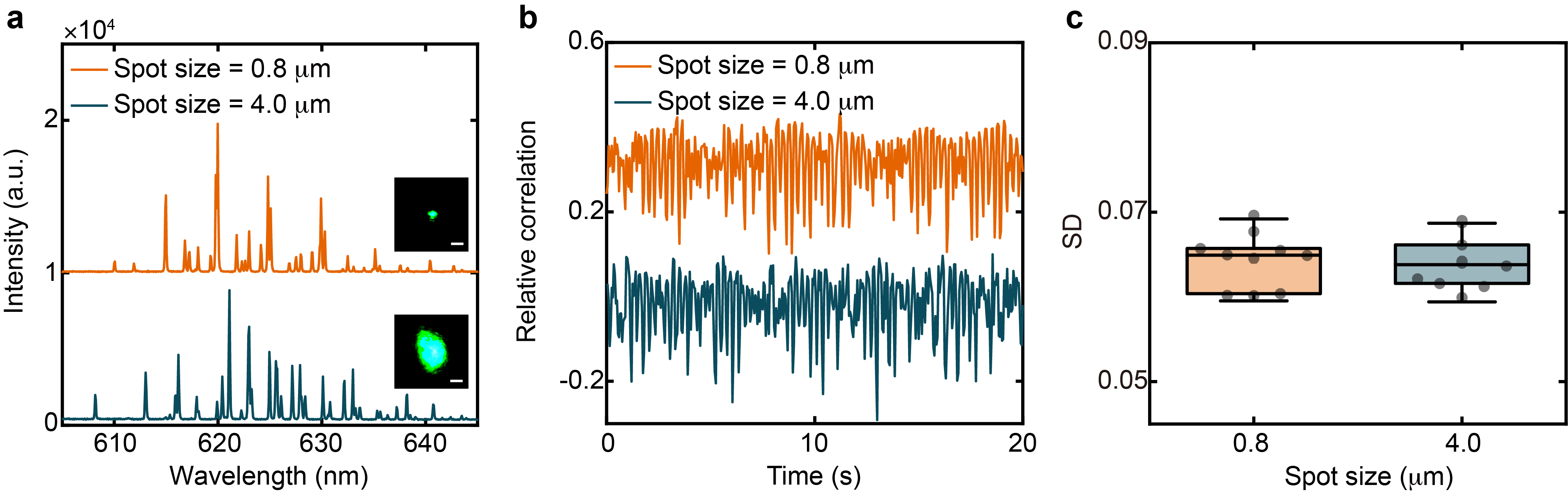


**Figure S18.** (**a**, **b**, **c**) the spectra (**a**), relative correlation curve (**b**), and SD (**c**) were obtained using different spot sizes. Inset, image of the pump spot. Scale bar: 1 µm. The error bars represent standard deviations of 10 times measurements.

## 8.4 Comparison of stage scanning and fixed measurement

We conducted additional experiments to investigate whether scanning affects the accuracy of viscosity measurement. Firstly, we compared the SD values obtained in different locations (Fig. S19). The result indicate that the location of pump spot has negligible influence on the viscosity measurement. This phenomenon can be explained by the relatively good symmetry of the microdroplet, which supports WGMs in all directions on the spherical geometry. Once the ultrasound induces mechanical vibration, the WGMs in all directions undergo similar time-dependent changes. Although the closed slit samples only certain regions of the droplet, similar SD can be obtained at different locations.


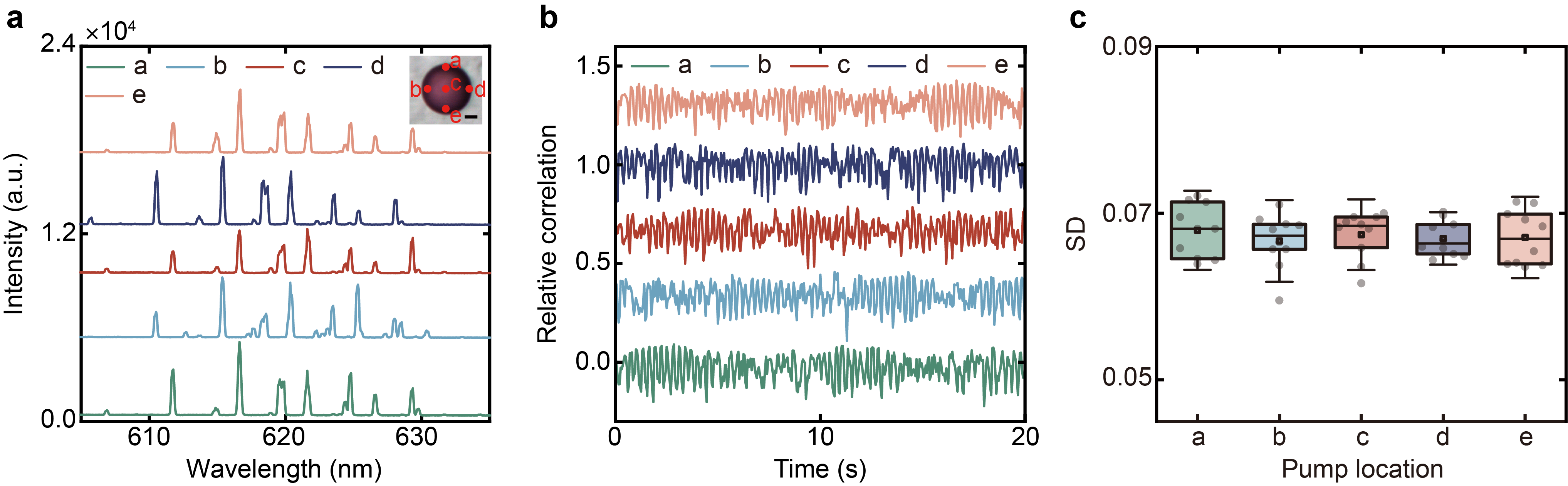


**Figure S19.** (**a**, **b**, **c**) the spectra (**a**), relative correlation curve (**b**), and SD (**c**) were obtained for pump located at different position of the droplet. Inset, image of the pump laser at different position of the droplet. Scale bar: 5 µm. The error bars represent standard deviations of 10 times measurements.

Secondly, we compared the time-resolved spectra, relative correlation, SD and FFT spectra obtained under fixed and scanning conditions. As illustrated in Fig. S20, stage scanning provides similar information to fixed measurement. Therefore, it can be concluded that stage scanning has no impact on the accuracy of viscosity measurement. In this experiment, the ultrasound signal with 132.4003 kHz and pump repetition rate (sampling frequency) of 1000 Hz were used.


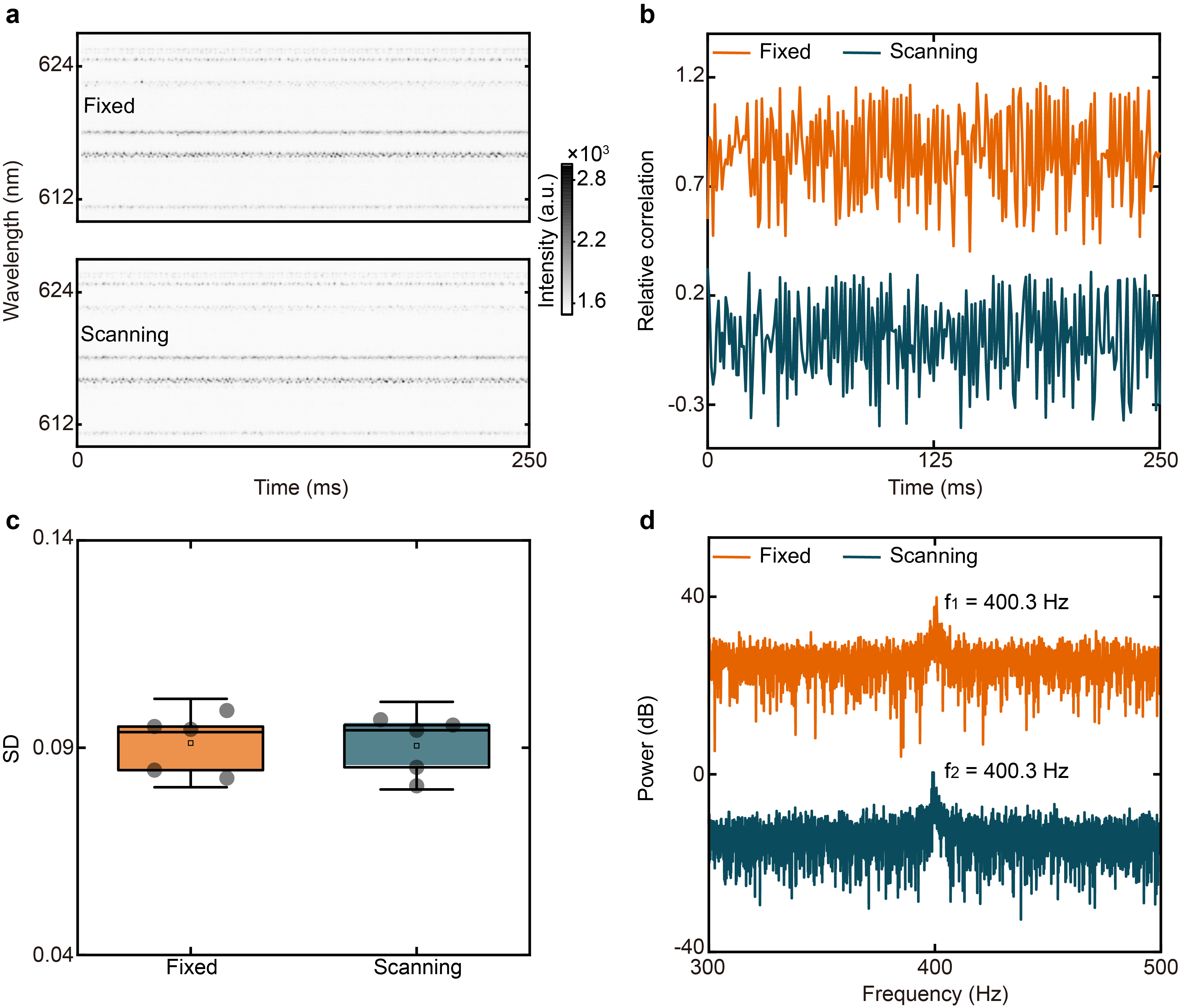


**Figure S20.** Comparison of time-resolved spectra (**a**), relative correlation (**b**), standard deviation (**c**), and FFT spectra (**d**) under fixed-stage and scanning conditions. Data are extracted from the same microdroplet. The error bars represent standard deviations of 10 measurements.

## 8.5 Stability of microdroplet size during scanning

As illustrated in Fig. S21, the free spectral range (FSR) of the WGM supported by the microdroplet is 4.22 nm. The diameter of microdroplet is calculated to be 20.75 µm by using $d={\lambda^{2}}/{(n\pi FSR)}$. Here, $n_{eff}$ = 1.398 is the refractive index. The temporal evolution of droplet size is given in Fig. S21b, no significant change in droplet size was observed during 120 min of ultrasound stimulation. A small diameter change of 1.8 nm was observed after 6000 laser pump excitations (Fig. S21d).


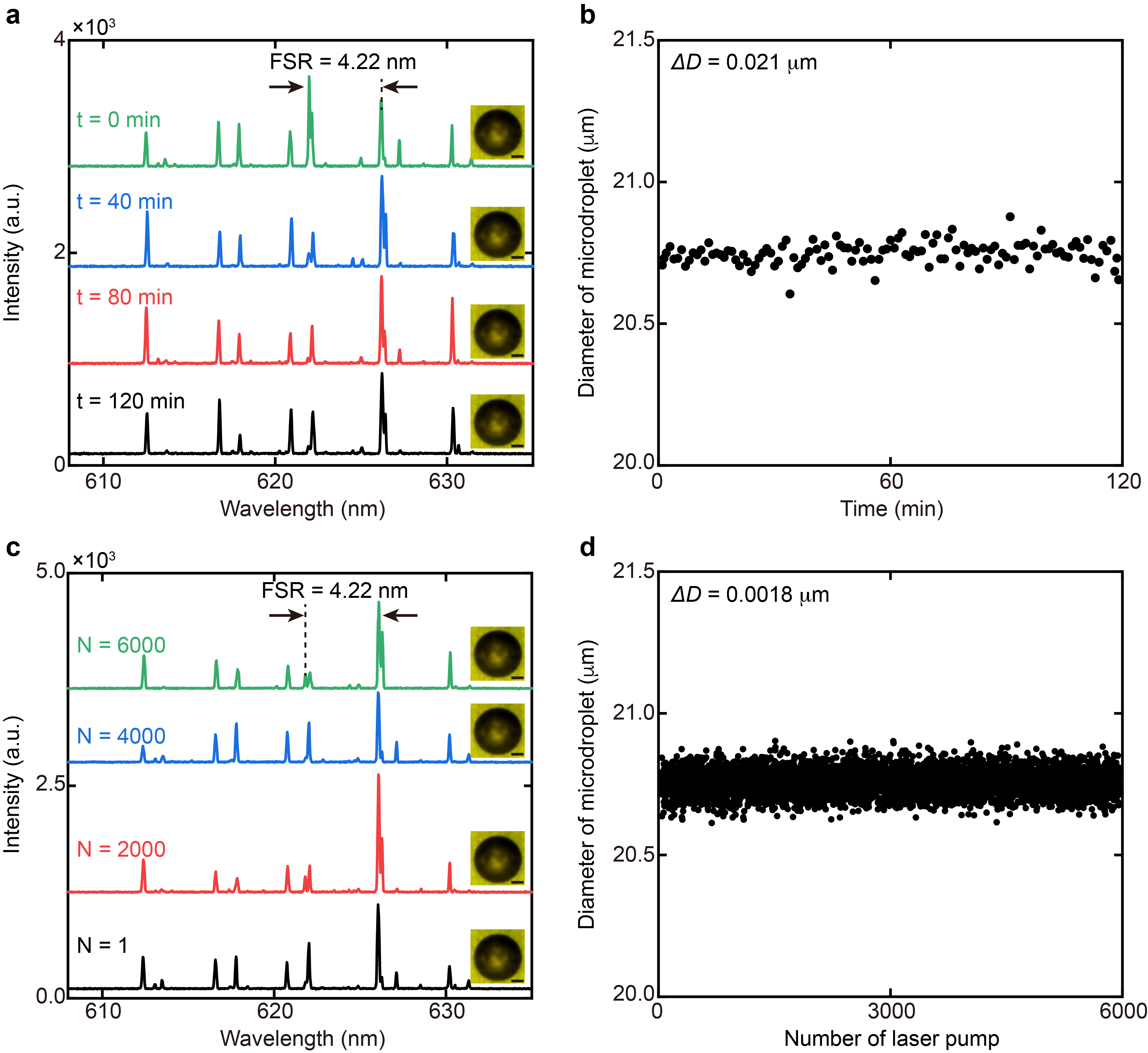


**Figure S21. (a)** The spectral and diameter (**b)** of microdroplet during 120 min of ultrasound stimulation. **(c)** The spectral and diameter (**d)** of droplet under different numbers of laser pump. Inset, image of the microdroplet. Scale bar: 5 µm.

# 9. Blood samples

We tested the viscosity of blood samples from four patient in high-throughput screening hyperlipidemia experiment. The total-cholesterol and triglyceride levels of different patients were shown in Table S2. More detailed procedure can be found in Methods.

**Table S2** Total cholesterol and triglyceride levels of different patients

| Patient | Total cholesterol (mM) | Triglyceride (mM) |
| --- | --- | --- |
| P1 | 2.73 | 0.63 |
| P2 | 4.73 | 0.66 |
| P3 | 5.15 | 1.85 |
| P4 | 6.13 | 11.05 |

# 10. Feasibility of clinic applications

We used tween 20 solutions with viscosity ranges from 3.5 to 9.5 mPa.s to test the feasibility of clinic applications, which covers the range of blood samples in Fig. 6. A linear fit between the measured results and the ground truth yielded (Table S3) a slope of 1.099, demonstrating a reliability of our method (Fig. S22).


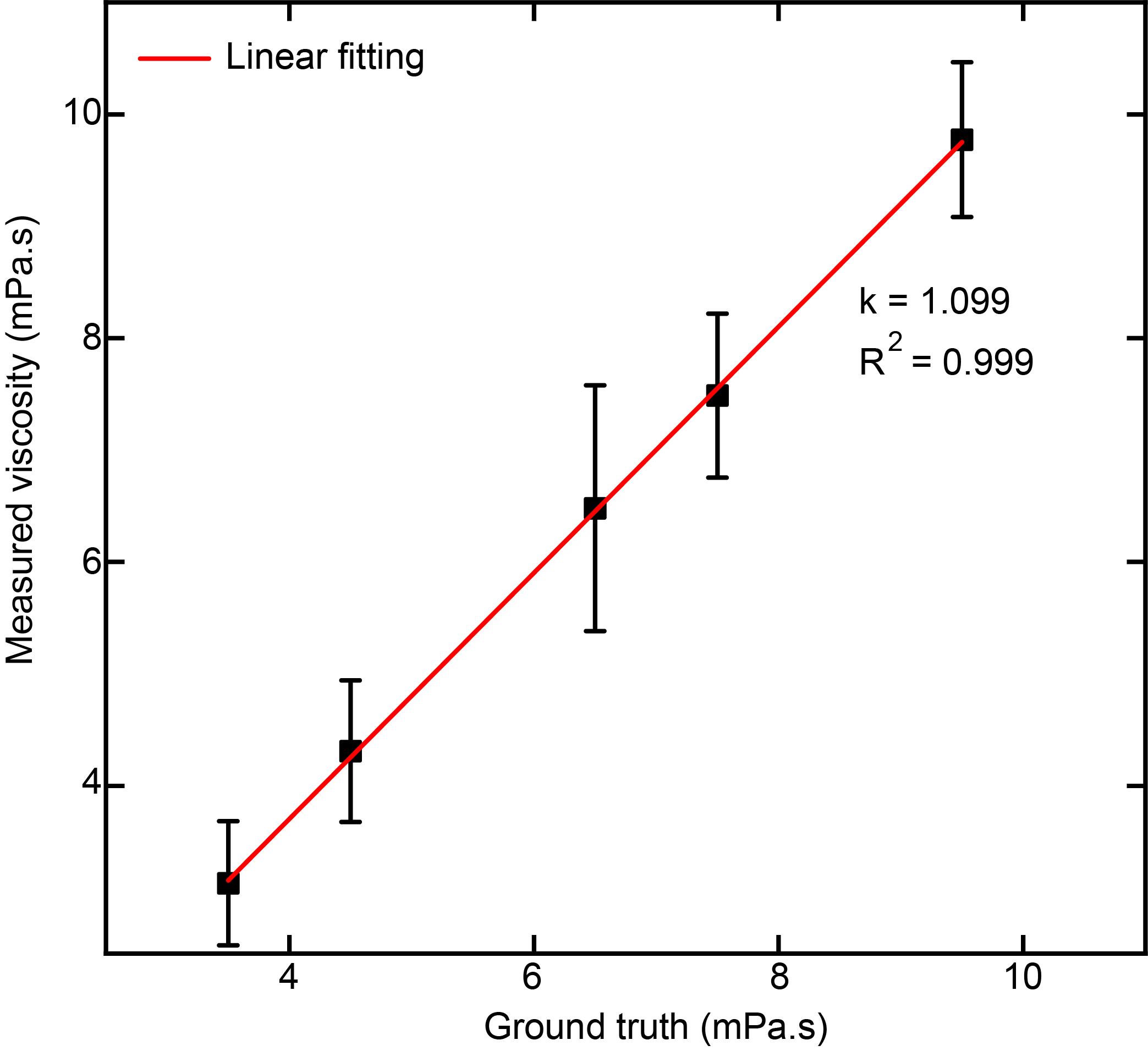


**Figure S22.** Feasibility of clinic applications

**Table S3** The ground truth viscosity and measured viscosity of the different tween 20 solutions

| Samples | Measured viscosity  (mPa.s) | Ground truth  (mPa.s)^12,14^ | Division |
| --- | --- | --- | --- |
| S1 | 3.13 | 3.5 | 10.57 % |
| S2 | 4.31 | 4.5 | 4.33 % |
| S3 | 6.48 | 6.5 | 0.31 % |
| S4 | 7.49 | 7.5 | 0.13 % |
| S5 | 9.77 | 9.5 | 2.84 % |

**Reference**

1 Moon, H. J. et al. Cylindrical microcavity laser based on the evanescent-wave-coupled gain. *Physical Review Letter* **85**, 3161-3164 (2000).

2 Kristoffersen, A. S. et al. Testing fluorescence lifetime standards using two-photon excitation and time-domain instrumentation: rhodamine B, coumarin 6 and lucifer yellow. *Journal of Fluorescence* **24**, 1015-1024 (2014).

3 Siegman, A. E. *Laser*. (University science books, 1986).

4 Dellwig, T., Lin, P. Y. & Kao, F. J. Long-distance fluorescence lifetime imaging using stimulated emission. *Journal of Biomedical Optics* **17**, 011009 (2012).

5 Miyamoto, K. et al. Nonlinear vibration of liquid droplet by surface acoustic wave excitation. *Japanese Journal of Applied Physics* **41**, 3465-3468 (2002).

6 Lyubimov, D. V., Lyubimova, T. P. & Shklyaev, S. V. Behavior of a drop on an oscillating solid plate. *Physics of Fluids* **18** (2006).

7 Zhang, S., Orosco, J. & Friend, J. Onset of visible capillary waves from high-frequency acoustic excitation. *Langmuir* **39**, 3699-3709 (2023).

8 Haus, H. A. Waves and fields in optoelectronics. (New Hersey: Prentice Hall, 1984).

9 Oppenheim, A. V. & Schafer, R. W. Discrete-time signal processing. 3rd edu. (Upper Saddle River: Prentice Hall Press, 2010).

10 Gregory, S. & Mach, H. Adaptation of a high-pressure liquid chromatography system for the measurement of viscosity. *Chromatography* **1**, 55-64 (2014).

11 Sharma, V. et al. Rheology of globular proteins: apparent yield stress, high shear rate viscosity and interfacial viscoelasticity of bovine serum albumin solutions. *Soft Matter* **7**, 5150-5160 (2011).

12 Szymczyk, K., Szaniawska, M. &Taraba, A. Micellar parameters of aqueous solutions of tween 20 and 60 at different temperatures: volumetric and viscometric study. *Colloids and Interfaces* **2**, 34 (2018).

13 Mandal, D. et al. Solvent induced morphological evolution of cholesterol based glucose tailored amphiphiles: transformation from vesicles to nanoribbons. *Langmuir* **32**, 9780-9789 (2016).

14 Nhaesi, A. H. & Asfour, A.-F. A. Prediction of the viscosity of multi-component liquid mixtures: a generalized McAllister three-body interaction model. *Chemical Engineering Science* **55**, 2861-2873 (2000).
